# Sampling strategies for genotyping common bean (*Phaseolus vulgaris* L.) Genebank accessions with DArTseq: a comparison of single plants, multiple plants, and DNA pools

**DOI:** 10.3389/fpls.2024.1338332

**Published:** 2024-07-11

**Authors:** Miguel Correa Abondano, Jessica Alejandra Ospina, Peter Wenzl, Monica Carvajal-Yepes

**Affiliations:** Genetic Resources Program, International Center for Tropical Agriculture (CIAT), Palmira, Colombia

**Keywords:** genotyping, sampling, genetic resources, common bean, DArTseq

## Abstract

**Introduction:**

Genotyping large-scale gene bank collections requires an appropriate sampling strategy to represent the diversity within and between accessions.

**Methods:**

A panel of 44 common bean (*Phaseolus vulgaris* L.) landraces from the Alliance Bioversity and The Alliance of Bioversity International and the International Center for Tropical Agriculture (CIAT) gene bank was genotyped with DArTseq using three sampling strategies: a single plant per accession, 25 individual plants per accession jointly analyzed after genotyping (*in silico–pool*), and by pooling tissue from 25 individual plants per accession (*seq-pool*). Sampling strategies were compared to assess the technical aspects of the samples, the marker information content, and the genetic composition of the panel.

**Results:**

The *seq-pool* strategy resulted in more consistent DNA libraries for quality and call rate, although with fewer polymorphic markers (6,142 single-nucleotide polymorphisms) than the *in silico–pool* (14,074) or the single plant sets (6,555). Estimates of allele frequencies by *seq-pool* and *in silico–pool* genotyping were consistent, but the results suggest that the difference between pools depends on population heterogeneity. Principal coordinate analysis, hierarchical clustering, and the estimation of admixture coefficients derived from a single plant, *in silico*–*pool*, and *seq-pool* successfully identified the well-known structure of Andean and Mesoamerican gene pools of *P. vulgaris* across all datasets.

**Conclusion:**

In conclusion, *seq-pool* proved to be a viable approach for characterizing common bean germplasm compared to genotyping individual plants separately by balancing genotyping effort and costs. This study provides insights and serves as a valuable guide for gene bank researchers embarking on genotyping initiatives to characterize their collections. It aids curators in effectively managing the collections and facilitates marker-trait association studies, enabling the identification of candidate markers for key traits.

## Introduction

Germplasm banks are repositories of crop genetic diversity. These collections include landraces, cultivars, wild forms, and closely related species. Not only do they serve a conservation purpose, but these plants and seeds are also a vital source of novel and underused genetic variation, an important input for national and private plant breeding programs to tackle the challenges faced by the agricultural sector (Byrne et al., 2018; Swarup et al., 2021). However, in the lengthy process of introducing novel genetic variation into a program, the first step requires field trials to identify candidates to start testing crosses with elite cultivars. This increases the cost of characterizing gene bank collections for complex traits like tolerance to abiotic stresses, considering that collections may number in the tens of thousands of accessions. To address this, multiple tools have been developed to improve the characterization of germplasm collections such as using passport and climate data to identify candidate accessions for abiotic stress tolerance (Smith et al., 1994; Greene et al., 1999; Cortés et al., 2013; Khoury et al., 2015; Haupt and Schmid, 2020).

As DNA sequencing and genotyping has become increasingly prevalent, they have been used to characterize germplasm collections of cultivated species worldwide. Examples include cowpea [*Vigna unguiculata* (L.) Walp.; Wamalwa et al., 2016], rice (*Oryza sativa* L.; Wang et al., 2018), forages [*Elymus tangutorum* (Nevski) Hand.-Mazz; Wu et al., 2019], cassava (*Manihot esculenta* Crantz; Adjebeng-Danquah et al., 2020), and common bean (Martins et al., 2006; Ariani et al., 2018; Nadeem et al., 2018). Emerging techniques have been developed, involving the use of one or more restriction enzymes to fragment genomic DNA, that enable the selection of specific genomic representations for subsequent sequencing and marker identification (Sansaloni et al., 2011). These advances significantly reduce the cost associated with genotyping numerous accessions. Nevertheless, genotyping thousands of plants still requires significant resources.

However, there is more to consider in a large-scale genotyping effort than just the sequencing strategy. A prime example is the seed bank of *Phaseolus* species conserved at the Genetic Resources Program of the Bioversity-CIAT Alliance (“the Alliance” or “ABC” hereafter). This remarkable collection encompasses approximately 38,000 plant materials, comprising all five cultivated species within the genus: the common bean (*P. vulgaris* L.), lima bean (*P. lunatus* L.), runner bean (*P. coccineus* L.), tepary bean (*P. acutifolius* A. Gray), and year bean (*P. dumosus* Macfady), along with approximately 40 wild species. The conventional practice of selecting a single random plant per accession for genotyping may not adequately represent the entire population (Gouda et al., 2020). This limitation arises because *Phaseolus* species exhibit a wide spectrum of mating behaviors, ranging from strictly allogamous to fully autogamous (Bitocchi et al., 2017). Moreover, there exists substantial variation within species themselves (Ibarra-Perez et al., 1997; Ferreira et al., 2000; Royer et al., 2002).

Genotyping more than 20–30 plants per population to obtain accurate allele frequencies and other population diversity estimates results in a significant increase (up to 30-fold) in genotyping costs, without accounting for additional space, labor, and time requirements. As a result, alternative sampling schemes are imperative for genotyping large collections. Pooling DNA has emerged as a promising alternative to individual sampling [for a review, see the work of Schlötterer et al. (2014)]. This approach involves the collection of equal volumes of plant tissue into a single tube, followed by a single DNA extraction for subsequent sequencing. Previous research has been conducted to explore the genetic diversity of various species using pooled data (Farahani et al., 2019; Ketema et al., 2020; Dziurdziak et al., 2021; Gapare et al., 2021; Arca et al., 2023). Recent comparative studies have investigated individual sampling with bulks of different sizes in rice (*Oryza* spp.) using DArTseq (Gouda et al., 2020), comparing whole-genome individual and pool sequencing of honey bee (*Apis mellifera* L.) (Chen et al., 2022) and studying the population structure of the American lobster with either GBS, rapture, or whole-genome pool-seq (Dorant et al., 2019). Despite research exploring the genetic diversity of species using pool data, little work has been done on the viability of pooling DNA from the common bean.

This study addressed this gap by using a diversity panel comprised of 44 accessions of the common bean (*P. vulgaris*) to compare two distinct sampling methods: individual sequencing or pooled sequencing. Our aim is to determine whether pooling DNA represents a viable alternative for studying the genetic diversity of the common bean gene bank collection. To achieve this, we evaluate how individual and pooled sequencing compare in terms of the number of markers identified through DArTseq, estimates of allele frequencies and heterozygosity, and the exploration of population structure of accessions of the species. This investigation contributes valuable insights into optimizing genotyping strategies for large-scale germplasm collections.

## Materials and methods

### Plant material and sample pooling

A total of 44 cultivated accessions of *Phaseolus vulgaris* L. were included in this study: 43 landraces and one modern cultivar (G4489; **Supplementary Table 1**). These accessions were selected from various continents including Africa, the Americas, Asia, and Europe. They were selected from the bean germplasm collection of the Alliance for the purpose of comparing the impact of pooling samples on allele frequency estimates. Thirty seeds from each accession were sown in the greenhouse at 25°C and 60% relative humidity at the ABC campus in Palmira-Colombia. Young leaf tissue was collected 15 days after sowing from each individual plant using a leaf tissue punch to obtain standard-size leaf discs. Tissue leaf discs were stored individually or pooled together in a single tube, to create the pool for each accession. All samples were stored at −80°C until DNA extraction. A total of 1,140 samples, including 1,096 individual samples and 44 pooled samples, were collected. The samples were intended to compare two types of pools: *seq-pools*, consisting of the 22 to 25 tissue leaf discs from individual plants collected in one tube for DNA extraction and sequenced as single samples per accession, and the *in silico–pools*, which comprise 22 to 25 individual plants each in single tubes for DNA extraction and sequenced independently. Subsequently, samples were analyzed together as *in silico*–pooled samples.

### DNA extraction, sequencing, and genotyping

Genomic DNA was extracted from around 10 mg of lyophilized leaf tissue from 2-week-old seedlings according to a modified Cetrimonium bromide (CTAB) protocol (Dellaporta et al., 1983; Doyle and Doyle, 1990). Extracted DNA was resuspended in 100 µL of TE buffer and incubated with 2 U of Ribonuclease (RNase) (40 µg/mL). DNA integrity was verified on a 0.8% agarose gel, whereas the quantity and purity were measured by calculating the absorbance at 260-nm/280-nm ratio using the Epoch spectrophotometer (Epoch). The final samples were then stored at −80°C until they were sent for sequencing. Samples were diluted to a final concentration of 50 ng/µL and were sent to Diversity Arrays Technology Pty, Ltd., Australia, for genotyping by sequencing with the DArTseq platform, using a medium-sequencing density (generating approximately 1.25 million reads per sample). In summary, a representation of the genomic DNA was obtained by digesting DNA with two restriction enzymes (*Pst*I and *Mse*I) and the prepared libraries were sequenced on an Illumina HiSeq2000 (Illumina). A total of 77 cycles were run to produce single reads. The reference-free marker calling step was done with a Diversity Arrays Technology Pty, Ltd (DArT P/L) proprietary method in the DS14 software. Reads were aligned to each other, with a threshold of two to three nucleotide mismatches, and used to call single-nucleotide polymorphisms (SNPs). Additionally, these reads were used to call presence/absence variations called SilicoDArT.

### Quality control and filtering loci

DArTseq SNP data csv files were read into R (V4.0.4; R Core Team, 2022) with the *gl.read.dart* function of the “*dartR*” package (V1.9.9.1; Gruber et al., 2018) and converted into genlight objects. Genlight objects were later split into three subsets: (i) one containing only individual samples and another, (ii) containing only pooled samples (*seq-pools*), and (iii) a single individual per accession (single plant).

**Figure 1:**
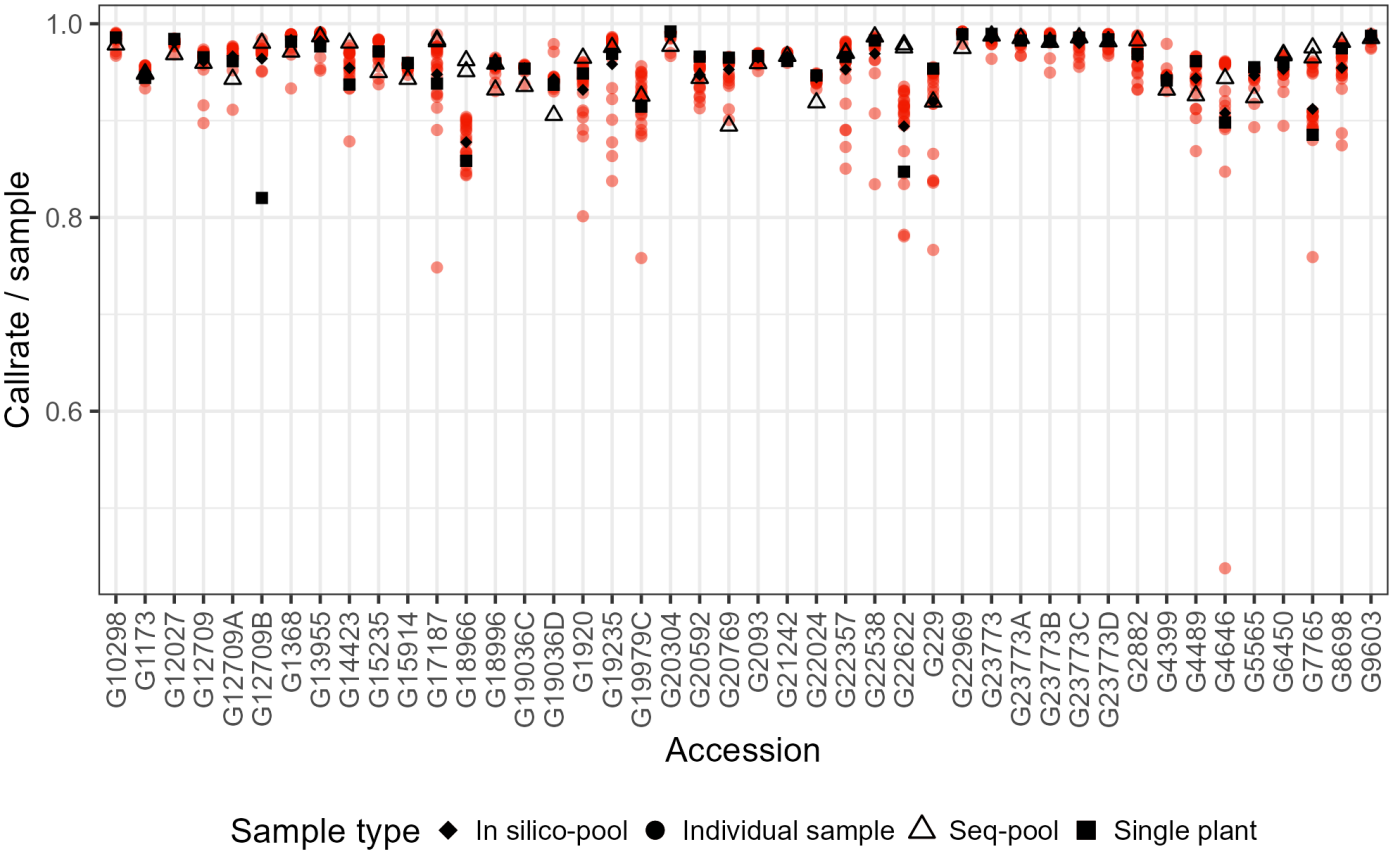
Comparison of the sample call rate between pools of *P. vulgaris* after filtering. Some accessions have two sequenced pools because of technical replicates.

A series of parameters were reviewed to identify potential samples and loci of low quality. This evaluation included the following: total reads per sample, total unique reads per sample, library quality (weak, downshifted, and good), sample call rate, loci call rate, minor allele frequency (maf), marker reproducibility, read depth, and polymorphism information content (**Figure 1**; **Supplementary Figures 1**-**4**).

Based on the descriptive statistics of the data, a set of filters was applied to all SNP subsets (individual samples and *seq-pools*) as follows: Replicability (RepAvg; the fraction of technical replicates at a locus with the same call) was set to 1; average read depth between 5 and 100 (as, unusually, high read depths can indicate paralogous regions of the genome mistakenly grouped together), and loci with call rate higher than 0.75 were retained (since samples cover a large geographical range, despite all belonging to the same species). Additionally, all monomorphic sites were removed from each dataset as they do not provide informative data.

To perform some estimations, we applied different filters. To estimate the expected heterozygosity (H_e_), the dataset of 1,086 individual samples was split by accession, all missing data within the subset was removed and, following the recommendations of the work of Schmidt et al. (2021), we estimated H_e_ before and after removing all monomorphic sites (for further details, see Data analysis section below).

An extra filter was incorporated to assess the resemblance between *seq-pools* and *in silico–pools* derived from the same accessions. This involved calculating the number of private alleles, the allele frequency difference (AFD), and a comparison of allele frequency estimates. Specifically, apart from the base filters mentioned above, an additional criterion was applied. Loci identified in both type of pools were retained by cross-referencing the AlleleIDs assigned by DArT P/L during the genotyping process. This additional step ensured a more rigorous comparison and enhanced accuracy of our analysis.

### Data analysis

#### Sampling a random individual

To evaluate the efficacy of pooled data (either *in silico–pools* or *seq-pools*) in comparison to genotyping a single individual per accession, a random sample of 44 individuals was selected from the larger dataset of 1,086 individuals, and this dataset will be referred as the single plant subset. Each individual was drawn from each accession using a custom R script (R Core Team, 2022) with a predetermined seed to ensure replicability. To see how sampling affected the data, 10 runs of the random sampling described above were performed. The identical set of filters mentioned previously was applied to this subset to maintain consistency in the analysis. All analyses were conducted across all three datasets, except for the estimation of allele frequencies, of H_e_, and the identification of private alleles (see below). Because the results from the 10 runs of sampling single plants were very consistent with each other, only the results of the first run are presented in the figures of the main text. The figures summarizing the results are available in the **Supplementary Material** (**Supplementary Figures 5**-**10**).

#### Allele frequency estimation and similarity between pools

To estimate the allele frequencies and assess the similarities between pools, we calculated allele frequencies within each accession for each kind of pool (*in silico–pools* and *seq-pools*). For *seq-pools*, DArT P/L provided an additional file alongside the standard report of SNPs and SilicoDArTs, containing the number of reads per allele per marker. Using these data, we calculated the frequencies as follows:


$$f_{ij}=\frac{\;\#reads_{ij}}{\sum_{j=1}^{2}reads_{j}}$$

where *f_ij_* is the allelic frequency of allele *i* at site *j*; # *reads_ij_* is the number of reads found for allele *i* at site *j*; and $\sum_{j=1}^{2}reads_{j}$ is the total number of reads at site *j*. Moreover, the allelic frequencies of *in silico–pools*’ SNPs were estimated on a per-accession basis by using the following formula:


$$p_{ij}=f\left(AA\right)+\;\frac{1}{2}f\left(AB\right);q_{ij}=1-p_{ij}$$

where *p_ij_* is the frequency of the reference allele *p* at locus *i* of accession *j*; *f*(*AA*) and *f*(*AB*) are the frequencies of the AA and AB genotypes, respectively; and *q_ij_* is the frequency of the SNP allele at locus *i* of accession *j*.

After estimating the allele frequencies of each dataset, we analyzed a series of key parameters within each pool. Specifically, we counted the number of called SNPs per pool, identified the number of missing sites, and determined the number of polymorphic sites within accessions.

To check the sampling effect on the estimate of allele frequencies, we used the technical replicates from DArT P/L for both *seq-pools* and single plants, assessing different read depth ranges. The average read depth per marker was estimated using the total read counts for the reference allele and the alternative allele, divided by the total of number of samples having reads for that marker. To compare the results from *seq-pools* and single plants derived from homogenous and heterogeneous accessions, we plotted the frequency of SNP allele reads at each marker across different read depth intervals.

#### Marker calling between pools and private and fixed alleles

To assess if there are differences between types of pools regarding the calling of markers, we compared the number of fixed and private alleles within each accession’s pool. Following rigorous filtering and quality control procedures (as detailed in Quality control and filtering loci section), we counted those sites where a pool exhibited an exclusive allele (referred to as a private allele) in comparison to the other pool. Additionally, we assessed sites where opposite genotypes were called in each pool (referred to as fixed alleles). This comparison aimed to highlight differences in allele calling patterns between *seq-pools* and *in silico–pools*. These counts of private alleles were fit to a generalized linear model specified as follows:


$$\log\left(p_{i}\right)=\eta_{i}=µ+\alpha_{i}$$

where log() is the logarithm link function between the linear predictor and the counts of private alleles (*p_i_*); µ is the general mean; and *α_i_* is the effect of the pool (*in silico–pool* or *seq-pool*). The model was applied using the “*glm*” function of R V 4.0.4 (R Core Team, 2022), utilizing the option “*family =* ‘*quasipoisson*’” due to identified overdispersion. This conclusion was drawn from a preliminary analysis where the ratio between residual deviance and degrees of freedom exceeded 1. The effect of the pool (*α*) was tested with an analysis of deviance, as implemented in the *Anova* function of the *car* package (V3.0–12) (Fox and Weisberg, 2019), utilizing the option “*test*.statistic = ‘F’.” The estimated means from the model were back transformed to the scale of the response variable using the *summary* function utilizing the option “*type =* ‘*response*’” in R.

In order to assess the similarity between allele frequency estimates across datasets, we calculated the AFD metric, as introduced by Berner (2019), which serves as an estimator of population differentiation to compare *in silico–pools*, *seq-pools*, and single plants. This measure was calculated using the following formula:


$$\text{AFD}=\frac{1}{2}\sum_{i=1}^{n}\left|f_{i1}-f_{i2}\right|$$

where *f_i_*_1_ and *f_i_*_2_ are the frequencies of allele *i* of an accession in datasets 1 and 2, respectively; and *n* is the number of markers.

#### Heterozygosity

Expected heterozygosity (H_e_) was calculated before and after the removal of monomorphic markers, following guidelines recommended by Schmidt et al. (2021). Schmidt et al. (2021) categorized these estimates as autosomal (considering all markers) and SNP (considering only polymorphic markers) heterozygosities. To avoid confusion, especially as the term “autosomal” implies a distinction from sex chromosomes, we have referred to these estimates as H’ [as per Schmidt et al. (2021)] and H for SNPs.

The H_e_ and H’_e_ were calculated using *in silico–pools* and the *seq-pools* dataset. The H_e_ was not estimated with the single plant dataset because this parameter is not commonly estimated on an individual basis, but rather on a population level, and we are working with accessions as populations. H_e_ (also known as gene diversity) is commonly defined as the expected frequency of the heterozygotes under Hardy-Weinberg equilibrium. Here, it was calculated as $H_{e_{i}}=2p_{i}q_{i}$ , where $H_{e_{i}}$ is the expected heterozygosity at site *i*, and *p_i_* and *q_i_* are the allelic frequencies at site *i*. Calculations of the estimates of the heterozygosity were made with custom R scripts.

#### Modified Roger’s distance and assessment of genetic patterns

The modified Roger’s distance (MRD) was calculated both between pairs of accessions within datasets and between samples of the same accession but different subsets. This calculation was based on matrices of allelic frequencies, each corresponding to a specific type of pool (Wright, 1978, p. 91). The pairwise distances were calculated as follows:


$${\text{MRD}}_{\text{xy}}=\frac{1}{\sqrt{2L}}\sqrt{\sum_{i\;=\;1}^{L}\sum_{j\;=\;1}^{2}{\left({\hat{p}}_{ij\left(x\right)}-{\hat{p}}_{ij\left(y\right)}\right)}^{2}}$$

where MRD*_xy_* is the distance between *x* and *y*; *L* is the number of SNPs in the dataset; ${\hat{p}}_{ij\left(x\right)}$ is the frequency of the *i*th allele at the *j*th locus of sample *x*; and ${\hat{p}}_{ij\left(y\right)}$ is the frequency of the *i*th allele at the *j*th locus of sample *y*. The matrices were calculated using a custom R script.

We employed various analytical techniques to unravel the genetic patterns within our dataset and to compare outputs across types of pools. Principal coordinate analysis (PCoA) was employed to understand the MRD matrix. PCoA, a dimensionality-reduction method, was executed using the “gl.pcoa” function from “dartR” package, generating a two-dimensional representation of the data. For clustering analysis, we utilized the complete linkage algorithm from the “stats” R package (V4.0.4) (R Core Team, 2022) to cluster the MRD matrix. The nodes of the resulting dendrogram were tested using a bootstrap analysis using the “boot.phylo” function of the “ape” package (V5.4.1; Paradis and Schliep, 2019) using parameters “rooted = FALSE” and “B = 1000.”.

To explore population admixture, we compared the best estimation of K ancestral populations derived from all individuals, the *seq-pools*, or a single individual per accession. This comparison was conducted using the “LEA” package and the “snfm” function in R (V3.2.0; Frichot and François, 2015). To run “snmf” with the *seq-pools*, the standard output from DArTseq was used because the input files for the “LEA” package are designed for allele counts, not allele frequencies. To run the analysis, the data (individuals, *seq-pools*, and single plants) as “genlight” objects were transformed into STRUCTURE input files using the “gl2structure” function of ‘dartR’ package (using option “exportMarkerNames = FALSE” and all others as default). The STRUCTURE-formatted files were then converted into the geno format through the “struc2geno” function of “LEA” (parameters; “ploidy = 2, FORMAT = 2, extra.row = 0, extra.column = 1”), facilitating further in-depth analysis of genetic admixture patterns. The “snmf” method from the “LEA” package was executed for each dataset with specific parameters: “K = 1:20, ploidy = 2, entropy = TRUE, CPU = 20, repetitions = 5, iterations = 500, alpha = 100.” The optimal K, indicating the most likely number of ancestral populations given the data, was determined using the cross-entropy criterion, selecting the point where the cross entropy exhibited a plateau. Initially the ‘snmf’ run with individual samples did not display a plateau, leading to an additional run with K-values from 40 to 55. Visual representations, including bar plots of admixture coefficients and cross-entropy values plots across different K-values were generated using the ‘ggplot2’ package (V3.3.3, Wickham, 2016).

## Results

Before applying any quality filters, a set of parameters, including total and unique read counts per sample, and the number of markers called, were assessed, and compared across different sample types.

For the 1,086 individual samples, the average total read count was 1,259,666 (± 211,597) and the average total unique read count was 201,500 (± 48,604). *Seq-pools*, consisting of 44 samples, exhibited a slightly higher average total and unique reads, reaching 1,271,141 (± 107,025) and 218,145 (± 21,432), respectively. In contrast, the 44 single plants showed the lowest mean counts of both total (1,241,579 ± 239,180) and unique (199,673 ± 53,303) reads along all the subsets. The counts of total and unique reads were more consistent across *seq-pools* samples (ranging from 985,347 to 1,443,516 and 167,534 to 267,046, respectively) than across individual samples (ranging from 594,075 to 1,744,258 and 91,370 to 364,280, respectively). The latter has a larger number of samples and a wider distribution across both variables, as reflected in the average and standard deviation of these counts on each dataset (**Table 1**).

**Table 1:** Summary of the comparison between pools before and after filtering.

Dataset	Variable		*In silico–pool*	*Seq-pool*	Single plant
General information	Number of accessions	–	44	44	44
	Number of samples	–	1,086	52	44
	Count of unique sequence reads per sample	Mean	201,500	218,145	199,673
		Std. dev.	48,604	21,432	53,303
	Count of total sequence reads per sample	Mean	1,259,666	1,271,141	1,241,579
		Std. dev	211,597	107,205	239,180
Unfiltered	Call rate/loci	Median	0.931	0.942	0.932
	Call rate per sample	Median	0.845	0.839	0.849
	maf	Mean	0.109	0.041	0.040
	Total number of SNPs	–	86,277	86,012	86,335
	Number of polymorphic SNPs across the dataset	–	31,677	15,453	15,340
Filtered	Call rate/loci	Median	0.983	1	0.977
	Call rate per sample	Median	0.963	0.969	0.951
	maf	Mean	0.110	0.241	0.240
	Number of polymorphic SNPs across the dataset	–	14,078	6,281	6,555

After splitting the SNP data by datasets (*seq-pools*, *in silico–pools*, single plants) and removing markers with 100% missingness, the total number of called markers was very similar among the unfiltered datasets from the three sample types: 86,012 in *seq-pools*, 86,277 in *in silico–pools*, and 86,335 in the single plant subset. Among these markers, 31,677, 15,453 and 15,340 were polymorphic, respectively. Notably, the *in silico–pools* exhibited a higher average of markers called per accession (78,427 SNPs ± 2,150.6) compared to either the *seq-pools* or the single plants, both of which had similar averages, 71,984 (± 2,634.5) and 71,909 (± 4,711), respectively (**Table 1**).

**Figure 2:**
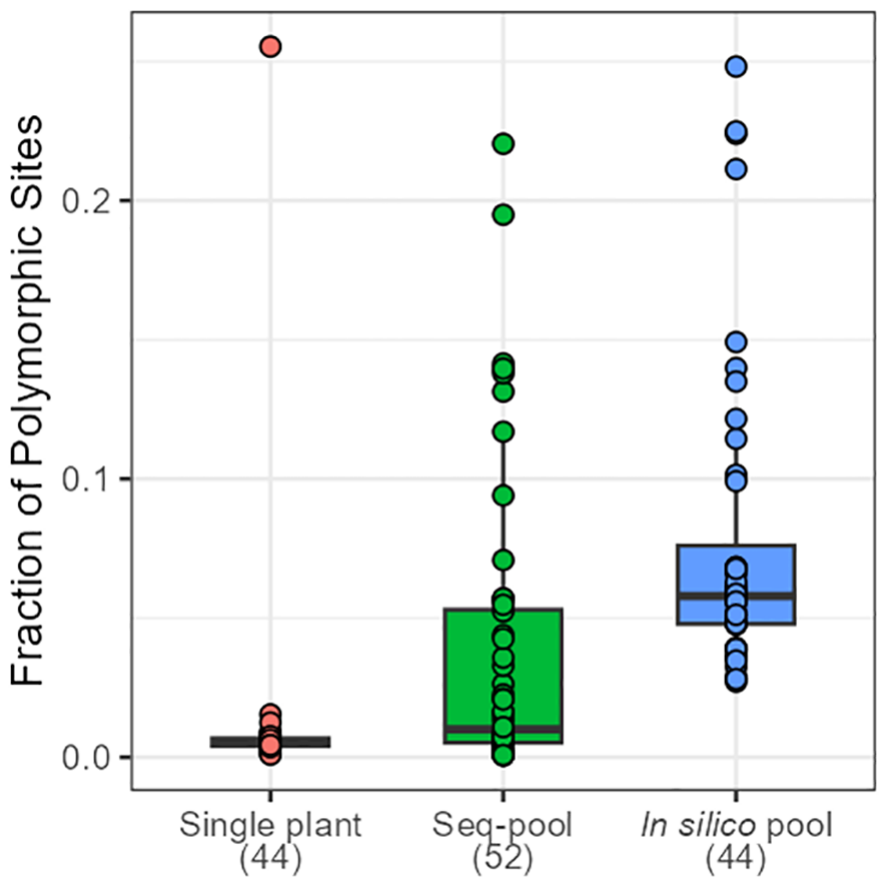
Distribution of the fraction of polymorphic SNPs across accessions of *P. vulgaris* on each dataset. Numbers in brackets indicate the number of samples per dataset.

The effects of applying a series of filters to remove SNPs (reproducibility = 1, average read depth 5–100, call rate/locus ≥ 0.75 and removing monomorphic sites) were assessed on based on call rate, number of polymorphic sites and allele frequencies estimates (**Table 1**; **Supplementary Figures 2**-**4**). After filtering, the number of remaining SNPs numbered 14,078 in the *in silico–pools*, 6,281 in the *seq-pools*, and 6,555 in the single plant datasets (**Table 1**). A comparison of the median call rate per sample showed similarity between *seq-pools (*0.963) and the individually genotyped samples (0.969), despite differences in the number of markers and the significant variation of call rates among samples from the same accession (**Figure 1**). The median call rate for the single plant subset was slightly lower at 0.951 (**Table 1**). The number of polymorphic sites per pool/single plant varied across each dataset. In general, the *seq-pools* tended to have fewer polymorphic sites than the *in silico–pools* from the same accession and slightly more than a single plant (**Figure 2**; **Supplementary Table 2**). The number of polymorphic sites ranged from 4 to 1,357 in *seq-pools*, 372 to 3,492 in the *in silico–pools*, and 5 to 1,582 in the single plant datasets. The distribution of polymorphic SNPs varied little across resampling runs for most of the accessions, while other accessions had outlier individuals (**Supplementary Figure 6**).

**Figure 3:**
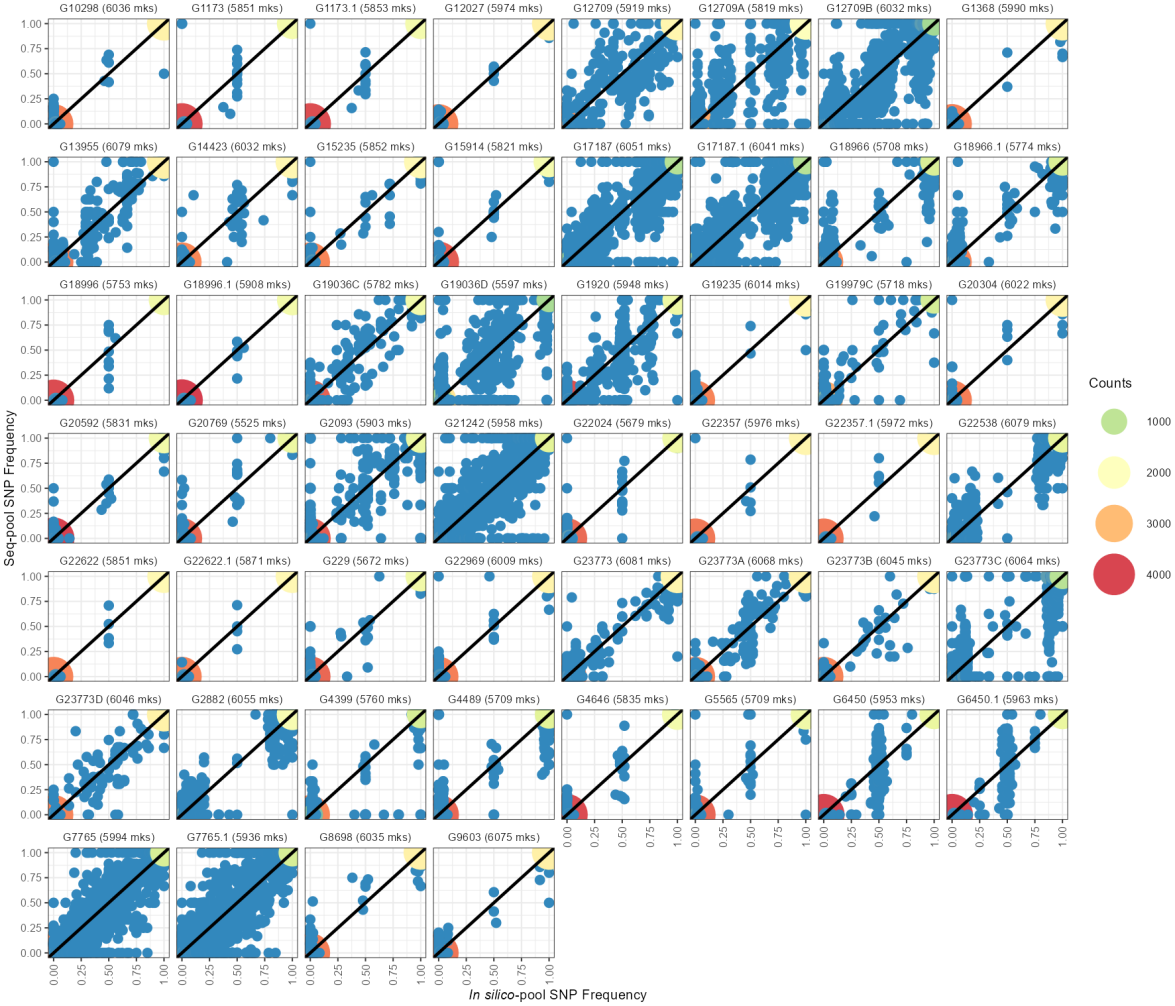
Comparison between allelic frequencies of the SNP allele between *in silico–pools* (X-axes) and *seq-pools* (Y-axes). Dot colors indicate the density of homozygotic sites for the same allele in both pools. Blue dots indicate heterozygote sites on either or both pools. Next to each accession ID is the number of shared markers between pools after filtering, including monomorphic SNPs.

The estimated allele frequencies from both pooled datasets revealed a wide range of homozygote markers within pools, from 75% to 97% in *in silico–pools* and 78% to 99.9% in *seq-pools* (**Figure 3**; **Supplementary Table 2**). Using the AlleleIDs from each pool type, we found that 6,142 (~97%) of the SNPs from the *seq-pool* data were also called in the *in silico–pools*. Comparing allele frequencies of these shared SNPs between types of pools showed that most markers coincide for the same allele in both pools (**Figure 3**). The distribution of the homozygous SNPs within *in silico–pools* showed two groups of accessions, one highly homogeneous (i.e., over 92% of homozygous SNPs) and one heterogeneous (<92% of homogeneous SNPs, **Supplementary Table 2**). When comparing the frequency of SNP allele reads estimated between available technical replicates (provided by DArT P/L) of *seq-pools* (e.g. G1173, G6450, G17187) it was observed that SNPs with an average depth below 20 reads had a higher discrepancy across replicates than SNPs with higher read depth. This trend was more evident in heterogeneous accessions (e.g. G17187). The frequency of SNP allele reads of single plants replicates, was more consistent between replicates (**Supplementary Figure 11**).

Some SNPs that were found to be monomorphic on one pool were polymorphic in the other, i.e., one of the pools had private alleles with respect to the other (**Figure 3**; **Supplementary Table 2**). After fitting a generalized linear model with a quasi-Poisson distribution, the analysis of deviance revealed a significant effect of the type of pool on the number of private alleles (Analysis of Deviance; Dev. Residuals = 24,221, DF = 1, F = 92.5, p-value = 5.694×10^-16^). The back-transformed estimated average of private alleles in *seq-pools* was 21.7, compared to an estimated 440.7 private alleles within *in silico–pools*. Fixed alleles (i.e., opposite alleles called in each pool) between pools were rare, for instance the highest observed count was 4 (**Supplementary Table 2**).

**Figure 4:**
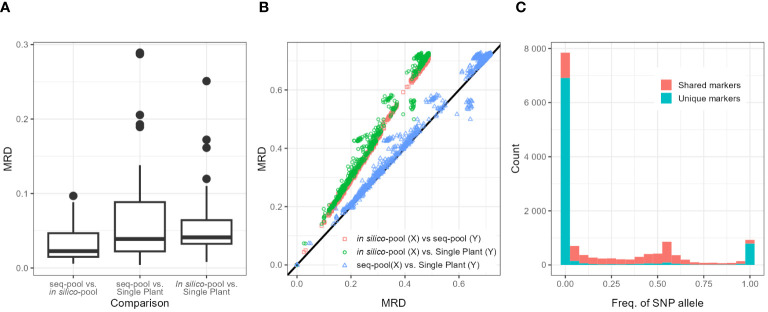
**(A)** Boxplots of the distribution of modified Roger’s distances (MRDs) between samples (*Seq-pool*, *in silico–pool*, or single plant) of the same accession. Labels on X-axis indicate comparisons. **(B)** Scatterplot comparing MRD matrices between pairs of datasets (*in silico–pool*, *seq-pool*, and single plant). Color of dots indicates datasets compared. Black line is the diagonal. **(C)** Distribution of SNP allele frequencies of the *in silico–pool* dataset, highlighting markers found only in that *dataset* (blue) and SNPs found in both *in silico–pool* and the *seq-pool* datasets (red).

The AFD is an estimator similar to F_st_ to measure differentiation between populations (Berner, 2019). The allele frequencies between the *in silico–pools* and the *seq-pools* two pools were highly similar, with a mean AFD of 0.008 (± 0.011) between pools. Accession G12709B, which showed a higher average AFD of 0.047 across shared loci, behaving as an outlier (**Supplementary Table 2**). Meanwhile, the MRD between both pools and the single plant of the same accession (**Figure 4A**) showed that the smallest distances were estimated between the pools (0.034 ± 0.026), while the distances of the single plants with either the *seq-pools* of the *in silico–pools* tended to be larger (0.066 ± 0.067 and 0.057 ± 0.047, respectively). When the data was split by homogeneous and heterogeneous accessions, the distances between *in silico–pools*, *seq-pools*, and single plants, tended to be smaller in the homogeneous group than in the heterogeneous group (**Supplementary Figure 12**). This pattern persisted even across all runs of resampling single plants (**Supplementary Figure 8**).

Although the shape of the distribution of the MRD (Wright, 1978, p. 91) was similar across datasets (**Figure 4B**; **Supplementary Figure 13**), the distances between *in silico–pools* were consistently smaller (Average MRD 0.341 ± 0.138) in comparison with either the *seq-pool* (Average MRD = 0.492 ± 0.203) or the single plant (Average MRD = 0.502 ± 0.209; **Table 2**). MRD was highly consistent across 10 runs of resampling single plants (**Supplementary Figure 7**).

**Table 2:** Summary (mean ± std. deviation) of the allele frequency difference (AFD) and the modified Roger’s distance (MRD) between accessions in each dataset.

Variable	*In silico–pool*	*Seq-pool*	Single plant
AFD	0.142 ± 0.087	0.313 ± 0.192	0.308 ± 0.193
MRD	0.341 ± 0.138	0.492 ± 0.203	0.502 ± 0.209

The difference among datasets was attributed to the presence of unique SNPs detected in the *in silico–pools* but not in the *seq-pools* which, as shown in **Figure 4C**, tend to be markers with very low frequencies. The distance matrix based on the 6,142 shared SNPs between the *in silico–pools* and *seq-pools* Showed an identical distribution to the *seq-pool* MRD matrix (**Supplementary Figure 13**). In contrast, estimating the distance matrix using markers exclusive to the *in silico–pool* data led to the lowest distances between *in silico–pools*, as shown in **Supplementary Figure 13** with “unique markers only.” A similar pattern was observed when the AFD was calculated (**Table 2**), i.e., the average similarity between *in silico–pools* was higher in this dataset (0.142 ± 0.087) than either the *seq-pool* (0.313 ± 0.192) or the single plant data (0.308 ± 0.193).

**Figure 5:**
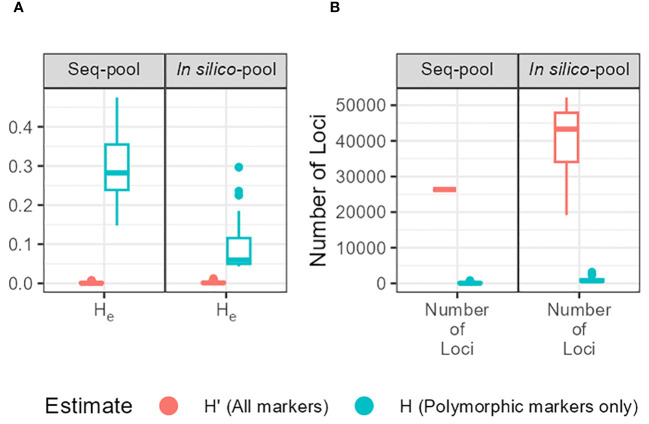
**(A)** Expected heterozygosity (He) estimates of each accession of *P. vulgaris* between types of pools. Estimates obtained with markers without missing data and either including monomorphic sites (H’; red) or not (H; blue). **(B)** Distribution of the number of sites per accession and type of pool used to calculate the estimates.

The gene diversity (*H_e_* = 2*pq*, expected heterozygosity) showed a significant variation between estimates (H_e_ and H’_e_) and between *in silico–pool* and *seq-pool* (**Figure 5**). The mean H_e_ was 0.0026 for the *in silico–pools* and 0.0017 with the *seq-pool* data. In contrast, H’_e_ was higher, averaging 0.09 and 0.31 in the *in silico–pool* and *seq-pool* datasets, respectively (**Supplementary Table 3**).

We employed SNP data and their corresponding distance matrices to investigate signs of population structure through PCoA, hierarchical clustering, and “snmf,” a method used to model admixture coefficients based on a given number of K ancestral populations.

**Figure 6:**
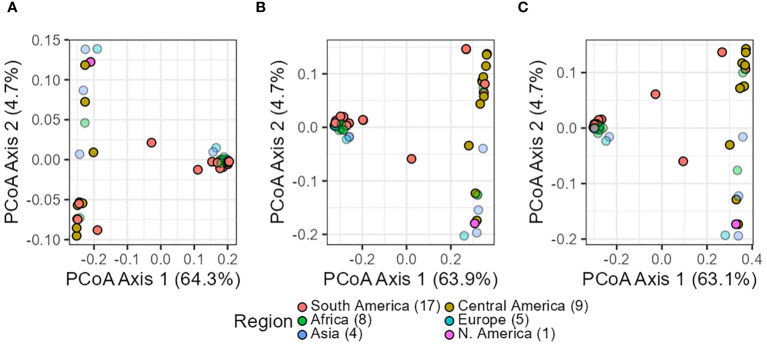
Scatterplots of the principal coordinate analysis (PCoA) after filtering the *in silico–pool* data **(A)**, the *seq-pool* data **(B)**, and the single plant subset **(C)**. Dot colors indicate origin according to passport data. Percentages in axes indicate proportion of the variance explained.

In summary, all three analyses yielded consistent results across datasets (*in silico–pools*, *seq-pools*, and a single plant). They uniformly revealed the divergence and separation between the Andean and Mesoamerican gene pools of common bean. For the PCoA, this distinction was evident in the first axis, explaining 63%–64% of the variance (**Figure 6**) and clearly separated accessions into two distinct groups. This applies as well to the 10 resampling runs of the single plant dataset (**Supplementary Figures 9**, **10**). Only two accessions, G21242 and G17187, were found in the space between the two groups, being more evident with the single plant subset (**Figure 6**). The second and third axes of the PcoA also showed an interesting pattern within each gene pool. Each axis split a group into two, with one composed mostly of accessions from the Americas and the other containing samples from other regions of the world (**Supplementary Figure 14**).

**Figure 7:**
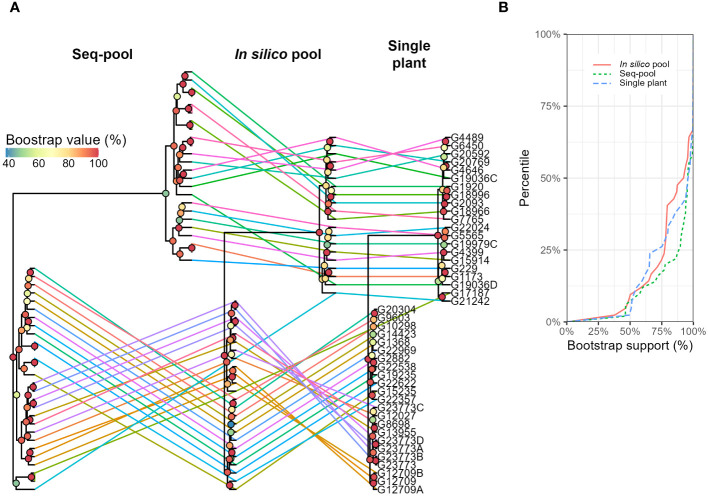
**(A)** Hierarchical clustering of 44 accessions of *P. vulgaris* using three different sampling types (*in silico–pools*, seq-pools, or a single plant). Lines and colors connect accessions across trees. Bootstrap shown with colors of nodes (n = 1000). **(B)** Distribution of the bootstrap support (%) for nodes of each dendrogram in **(A)**.

The hierarchical clustering analysis also separated two larger groups (**Figure 7A**). Although smaller groups were inconsistent, with low bootstrap support (< 75%; **Figure 7B**). Whereas most accessions remained within the same two major clusters across the three sampling types, two accessions, G21242 y G17187, exhibited differential clustering patterns in *seq-pools* compared to *in silico–pools* and a single plant. Moreover, eight replicated *seq-pools* used by DArT P/L to estimate the replicability of the marker calling steps were also included into the tree and they confirmed the robustness of the clustering by being consistently groups together with their replicates (**Figure 7A**; *Seq-pool*). The panel of this study included three accessions that were subdivided into multiple accessions over time: G12709 (three accessions), G19036 (two accessions), and G23773 (five accessions). Of these, only G12709 was consistently clustered together across all trees (**Figure 7A**).

**Figure 8:**
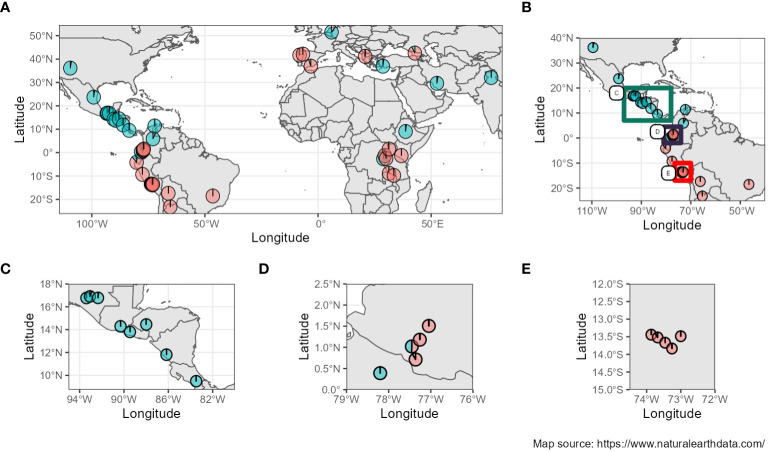
**(A)** Admixture coefficients at K=2 from *seq-pool* data mapped according to coordinates of origin from the accessions’ passport data. **(B–E)** Close-ups of the American continent (Passport information source: https://www.genesys-pgr.org/a; Map source: https://www.naturalearthdata.com/).

**Figure 9:**
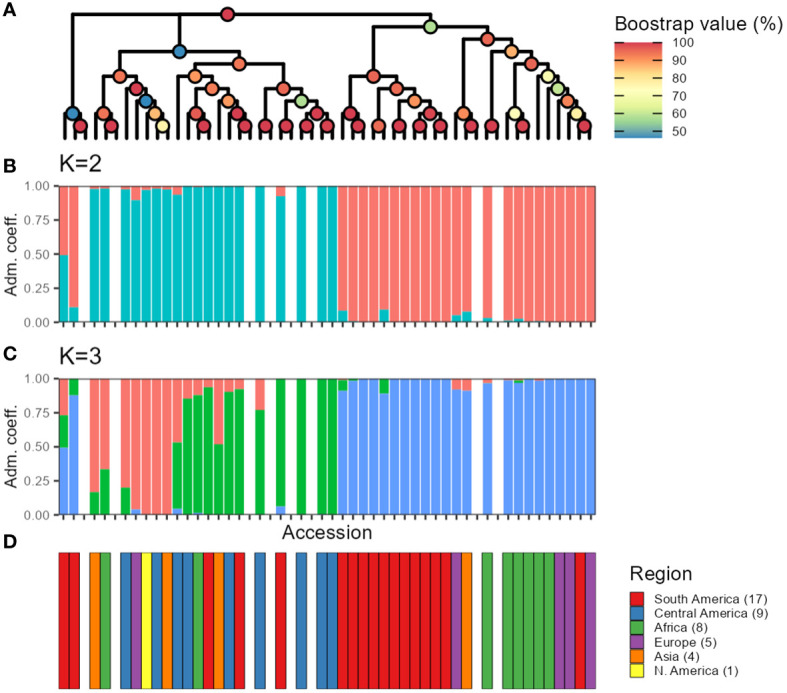
Comparison between hierarchical clustering using the sequenced pools estimated allele frequencies **(A)**, and snmf using allele counts **(B, C)**. The color of the nodes in **(A)** indicates bootstrap support (n = 1,000). Colors of the bars in **(B, C)** indicate fraction of the genome presumed to originate from different ancestral populations. **(D)** Region of origin of the accessions. Missing bars in **(B-D)** indicate technical replicates from DArTseq that were included when making the tree in **(A)**.

Furthermore, when studying the admixture coefficient of ancestral populations, the best fitting K-value accordingly to “snmf” was K = 2 for both the *seq-pool* and the single plant data, with a cross-entropy of the best run at 0.40 (**Supplementary Figure 15**). When mapping the admixture coefficients of the *seq-pool* data using the accessions’ passport data, the distribution of the ancestral populations across the Americas has a clear north-south split. That is, most Accessions originating to the south of Ecuador shared the same ancestral population, whereas accessions distributed across Central and North America shared the other ancestral population in common (**Figure 8**). Regarding the accessions from Africa, Asia, and Europe, most seem to share the same ancestral population with that of the South American accessions, but no clear pattern could be discerned (**Figure 8A**). These results are highly consistent with the two large clusters found with the hierarchical clustering (**Figure 9**).

## Discussion

In the last decade, there is been a notable increase in genomic characterization of long-preserved collections (Wang et al., 2018; Sansaloni et al., 2020). This trend is driven by cheaper sequencing costs and the increasing focus on maximizing the value of each accession in germplasm collections. The genetic data acquired offers valuable insight to curators, aiding decision-making and improving access to alleles and genes linked to key traits. However, challenges persist, particularly in determining optimal sampling methods. Balancing the need for representing accessions or populations with cost-effectiveness is especially crucial for large germplasm collections managed by CGIAR. Achieving the right balance between scientific rigor and practicality is essential for effectively navigating these challenges. In this study, we genotyped 44 accessions of *P. vulgaris* using three sampling strategies to assess if analyses based on the genotype calls, estimated allele frequencies, diversity estimates, and population structure yielded consistent results across sampling methods. Our findings indicate that *in silico–pools* yielded a higher number of SNPs compared to both *seq-pools* and the single plant data. This is attributed to the individual genotyping of each member within the *in silico–pool*, which increases the likelihood of identifying rare alleles. However, calling SNPs from pooled DNA samples poses a challenge in distinguishing genuine rare variants from sequencing errors (Schlötterer et al., 2014; Anand et al., 2016). Similarly, there remains uncertainty when sampling a random individual per population/accession, as it may not accurately represent the entire population. Filtering and handling missing data are critical in genetic analyses. Methods have different tolerances to missing data, and strict filters can negatively impact downstream inferences (Wiens, 2006; Rubin et al., 2012; Huang and Knowles, 2014; Eaton et al., 2017). Conversely, some methods struggle when missingness is non-random, depending on factors like species or gene pools (Yi and Latch, 2022).

The overall population patterns observed in PCoA, snmf, and the hierarchical clustering across datasets (*seq-pool*, *in silico–pool*, and a single plant) after applying uniform filters (Reproducibility = 1, average read depth = 5–100, call rate/locus ≥ 0.75, no monomorphic sites) were similar. While these criteria may appear “lax” compared to general recommendations for filtering marker data (e.g., Carson et al., 2014; O’Leary et al., 2018; Pavan et al., 2020), our dataset encompasses a wide range of samples from diverse geographic origins, each subjected to different selection pressures and accumulating genetic differences. Similar lax filters have been employed in other studies investigating common bean genetic diversity (Valdisser et al., 2017; Nadeem et al., 2020; Gelaw et al., 2023). In this work, the aim was to retain sites displaying allele dropout, a common challenge in reduced representation approaches like DarTseq (Gautier et al., 2013), as they provide valuable insights information where they are present, making them informative across diverse populations (Wiens, 2006). Thus, imputation was not performed to avoid assumptions about the cause of missing markers, acknowledging the biological nature of allele dropout.

Accurate estimation of allele frequencies is crucial, as it directly influences MRD matrices. While using single plants poses challenges due to varying call rates within an accession and potential bias from missing data (as depicted in **Figure 1**). Studies have found that estimating allele frequencies with pooled data can be more precise. This is attributed to reduced DNA contribution variance, particularly with larger pool sizes (Futschik and Schlötterer, 2010; Rellstab et al., 2013). In our study, comparing allele frequencies between *seq-pools* and the *in silico–pools* revealed low AFD, suggesting minimal differentiation between pools of the same accession. Although allele frequency estimates from *seq-pools* and *in silico–pools* appear correlated, the large sample size and counts of fixed markers consistently return a strong and significant correlation every time, which is why they are not shown here.

*Seq-pools* exhibit limitations in estimating intermediate (~0.5) frequencies (**Figure 3**), regardless of the population’s polymorphic loci count. Theoretical and empirical research indicates that variance and error of allele frequencies are highest at intermediate frequencies (Chen et al., 2012; Fung and Keenan, 2014), as is the difference between simulated and empirical allele frequencies (Hale et al., 2012). Other causes include technical artifacts such as random amplification of reads, insufficient locus depth, or uneven DNA contributions. The latter is unlikely due to meticulous control of sample tissue area per plant for consistency across individuals. When we compared the frequency of SNP allele reads between technical replicates, we observed that the least consistent estimates of allele frequencies were found in the SNPs with average depth<20 reads (**Supplementary Figure 11**). This difference between replicates was more evident in heterogeneous accessions for seq-pools that from single plants, suggesting a sampling effect on seq-pools, most likely due to random amplification of reads during library preparation or insufficient locus depth of rare alleles from individual including in the pool. Although a similar pattern is seen in replicates of single plants, the frequency of SNP allele reads is more consistent.

Another possible cause could be the method of allele frequencies estimation from pooled data, known as the “naive” method, where allele reads’ ratio at a locus serves as the estimate [as used by Inbar et al. (2020)]. This method may inflate minor allele frequency estimates, particularly for rare alleles (Chen and Sun, 2013). While tools exist for calling markers with pooled DNA data [see the work of Schlötterer et al. (2014) for a list of methods and for an in-depth comparison between callers], these pipelines require aligning reads to a reference genome (Guirao-Rico and González, 2021). To our knowledge, this is the first instance where read count data from DArTseq has been used for estimating allele frequencies. Regular allele counts from pools of different sizes have been employed in other crops such as Barley (*Hordeum vulgare*; Dziurdziak et al., 2021), chickpea (*Cicer arietinum*; Farahani et al., 2019), cowpea (*Vigna unguiculata*; Ketema et al., 2020), pastures (*Phalaris aquatica*; Gapare et al., 2021), and safflower (*Carthamus tinctorius*; Hassani et al., 2020).

Overall, both *in silico*– and *seq-pools* exhibited high similarity, evidenced by the low AFD, minimal private alleles between pairs, and genetic distances (**Figures 4A, B**; **Supplementary Table 2**). Despite that *in silico–pools* do discover more markers (**Supplementary Table 2**), predominantly low-frequency SNPs (**Figure 4C**), the overall difference between pools of the same accession was small. However, sample similarity was also influenced by the within-population diversity, as heterogeneous accession groups revealed higher MRD between samples of the same accession (**Figure 3**; **Supplementary Figure 11**), potentially indicating single plants’ insufficient representation of an accession. Regarding the single plant datasets, consistency across multiple random sampling runs was observed (**Supplementary Figures 5**-**10**) and with either the *seq-pool* or *in silico–pool* data (**Figure 7**). Nevertheless, a significant discrepancy was noted in the number of detected SNPs in this dataset (**Figure 2**; **Supplementary Figure 6**, **Supplementary Table 2**), suggesting that single plant data underestimates within-accession variation, which is crucial for comprehending species diversity.

After SNP filtering across datasets, a notable disparity in the count of polymorphic sites within accessions was observed. For instance, the variance in polymorphic markers between *in silico–pools* of accessions G12709B and G20592 was substantial, with 3,492 vs. 372 SNPs, respectively. This difference was even more evident in the *seq-pool* data, with counts of 1,357 vs. 32 SNPs, respectively. In contrast, the difference between single plants of these accessions was minimal, with 16 vs. 9 SNPs (**Supplementary Table 2**). When examining gene diversity (expected heterozygosity, H_e_) across *in silico–pool* or *seq-pool* data, the H’_e_ estimates suggest that certain populations harbor minor alleles with moderate to high frequencies, indicating potential population sub-structure or outcrossing events. Conversely, the H_e_ estimates derived from either pooled dataset present a nuanced view of accession diversity across our panel. Although H_e_ varies considerably across populations/accessions, the values remain quite small (ranging from 0.0004 to 0.0128 for *in silico–pool*’s data and from 0.000034 to 0.008151 for *seq-pool*’s data), which fits better with a species that is mostly self-pollinating. Estimating H_e_ based on single plant dataset would not accurately represent the entire accession. Furthermore, the distribution of genetic distances was notably influenced by the presence of low-frequency alleles. Although the shape of the distribution across all datasets appeared similar (refer to **Supplementary Figure 13**), the distance matrix derived from the *in silico–pool* data was consistently smaller in magnitude (**Table 2**). This difference between datasets nearly disappeared when shared markers between pools were used to calculate genetic distances (**Supplementary Figure 13**). The presence of low-frequency SNPs reduces the MRD by increasing the denominator (2N) in the MRD formula (see Materials and Methods). Similarly, the AFD distribution was comparable between the *seq-pool* and the single plant data (**Table 2**), whereas the *in silico–pool* data displayed greater similarity between accessions, indicating that this metric is also sensitive to a substantial fraction of very rare alleles.

Variation in the within-population diversity of landraces of common bean was observed (**Figures 3**, **5**), potentially attributed to the diverse origins of the included accessions in this study (**Supplementary Table 1**) and the fact that landraces are generally more genetically diverse compared to modern counterparts (Byrne et al., 2020; Wilker et al., 2020). Across the accessions included in this study, there are some homozygous accessions for almost all loci with some residual heterozygosity (e.g., G10298 and G1368), whereas other accessions are more heterozygous (e.g., G17187 and G21242). The more heterogeneous accessions suggest that they could be a mixture of seeds, a frequent scenario in common bean, potentially enhancing diversity (Blair et al., 2010; García-Narváez et al., 2020). This contrasts with the expected low within-population diversity of a mostly selfing species like *P. vulgaris*, noting that crossing rates may vary from 2.5% up to 70% (Wells et al., 1988; Ibarra-Perez et al., 1997; Ferreira et al., 2000; Royer et al., 2002; Chacón-Sánchez et al., 2021). While DNA pooling is uncommon in common bean genetic diversity studies, its application has focused on variations between gene pools (Papa et al., 2007) or used in different marker systems like microsatellites (Zhang et al., 2008; Asfaw et al., 2009) and simple sequence repeats (Özkan et al., 2022). Because the most diverse accessions coincided between *seq-pools* and *in silico–pools* (**Supplementary Table 2**), *seq-pools* offers a promising approach for identifying accessions with high genetic diversity (heterogenous accessions). This information is valuable not only for gene bank users but also for seed collection curators. This highlights a limitation of single plant data because one individual may not adequately represent the diversity of an entire population/accession. This limitation is particularly relevant in the study of landraces, wild forms of *P. vulgaris*, and cross-pollinating *Phaseolus* species. After all, fewer polymorphic SNPs were detected within accessions compared to both *seq-pool* and *in silico–pool* data, emphasizing the importance of pooled sequencing methods for comprehensive diversity assessment (**Figure 4A**; **Supplementary Table 2**).

Apart from the mentioned challenge of estimating allele frequencies, a key limitation associated with the use of *seq-pools* lies in the difficulty in accurately estimating the observed heterozygosity within populations of an accession, as highlighted previously by Chen et al. (2022). In our study, we were unable to compare estimates of H_o_ across datasets. This metric can only be calculated using the *in silico–pools* dataset, where individual genotypes are available and not with *seq-pools* or single plants. Additionally, pooling does not allow us to distinguish whether a heterogeneous accession results from a recent cross or a seed mixture.

As mentioned above, PCoA, hierarchical clustering, and “snmf,” revealed consistent patterns of population structure within *P. vulgaris*, identifying two major ancestral groups across all datasets: *seq-pool*, *in silico–pool*, and single plant datasets. These findings align with the current consensus of domesticated *P. vulgaris* having two major gene pools: the Mesoamerican and the Andean groups (Blair et al., 2012). We also identified G21242 as a potential hybrid, consistent with previous research (Blair et al., 2006) Our results parallel the findings of Arca et al. (2023) in maize pools, demonstrating the consistency of PCoA, hierarchical clustering, and admixture coefficients, albeit utilizing microarray and measurement of fluorescence ratios data for allele frequency estimation. Whereas the PCoA and the hierarchical clustering exhibited similar patterns across datasets, the PCoA based on allele frequencies from *seq-pool* data revealed more distinct groups along the second and third axes compared to *in silico–pool* or single plant data (**Supplementary Figure 14**). Notably, the division within major groups appeared to segregate American and non-American accessions, which could be attributed to the selection process after introduction into new environments. The “snmf” analysis with *in silico–pool*’s utilized all 1,086 individual samples, leading to a significant difference in estimating the optimal number of ancestral populations compared to *seq-pool* and the random individual data (**Supplementary Figure 15**). This discrepancy could be attributed to data redundancy or a bias from abundant rare alleles with low informativeness (Linck and Battey, 2019). Conversely, the analysis with the *seq-pool* samples showed less sensitivity, possibly due to the smaller number of accessions studied (n = 44), which may not have sufficient for rare alleles to exert significant influence. Nevertheless, the *seq-pool* data remained highly consistent with the estimated ancestry coefficients derived from *in silico–pools* and single plants at K = 2 (**Supplementary Figure 16**).

Our findings demonstrate that using pooled DNA for studying the genetic diversity of domesticated *Phaseolus vulgaris* yields comparable insights to sequencing individuals, despite certain limitations such as challenges in estimating intermediate allele frequencies and lack of individual genotypes. Despite these limitations, pooled samples remain the most practical sampling strategy for large-scale genotyping efforts of germplasm collections. Genotyping individuals significantly multiplies the workload and resources required by a factor of “n” (where “n” represents the number of samples to be pooled). This increased demand extends not only to field and lab work but also to sequencing efforts, genotyping, and all subsequent data analyses, requiring substantially larger computational resources and processing time. Although other alternatives, such as WGS or arrays, exist to genotype plant genetic resources, the former remains costly for large-scale projects, although it has the advantage of generating significantly more data. Microarrays, on the other hand, have well-known issues with ascertainment bias (Arca et al., 2023), and the amount of data generated would be insufficient for association studies or analyses beyond genetic diversity.

This study provides valuable guidance for gene bank researchers undertaking genotyping initiatives, aiding in effective collection management, and facilitating marker-trait association studies for identifying candidate markers associated with key traits.

## Data availability statement

The data presented in the study are deposited in the Dataverse repository, accession number https://doi.org/10.7910/DVN/MQCSC4.

## Author contributions

MCA: Data curation, Formal analysis, Visualization, Writing – original draft, Writing – review & editing, Methodology. JO: Formal analysis, Methodology, Writing – review & editing. PW: Conceptualization, Methodology, Supervision, Writing – review & editing, Funding acquisition. MC-Y: Methodology, Supervision, Writing – review & editing, Conceptualization, Project administration, Writing – original draft.
