# Supplementary material for: Sampling strategies for genotyping common bean (Phaseolus vulgaris L.) Genebank accessions with DArTseq: a comparison of single plants, multiple plants, and DNA pools
Source: Front Plant Sci. 2024 Jul 11;15:1338332. doi: 10.3389/fpls.2024.1338332 (PMC11269218; doi:10.3389/fpls.2024.1338332)
Supplement: Supplementary file 1 [file DataSheet_1.docx]

Supplementary Material

# Supplementary Figures and Tables

## Supplementary Figures


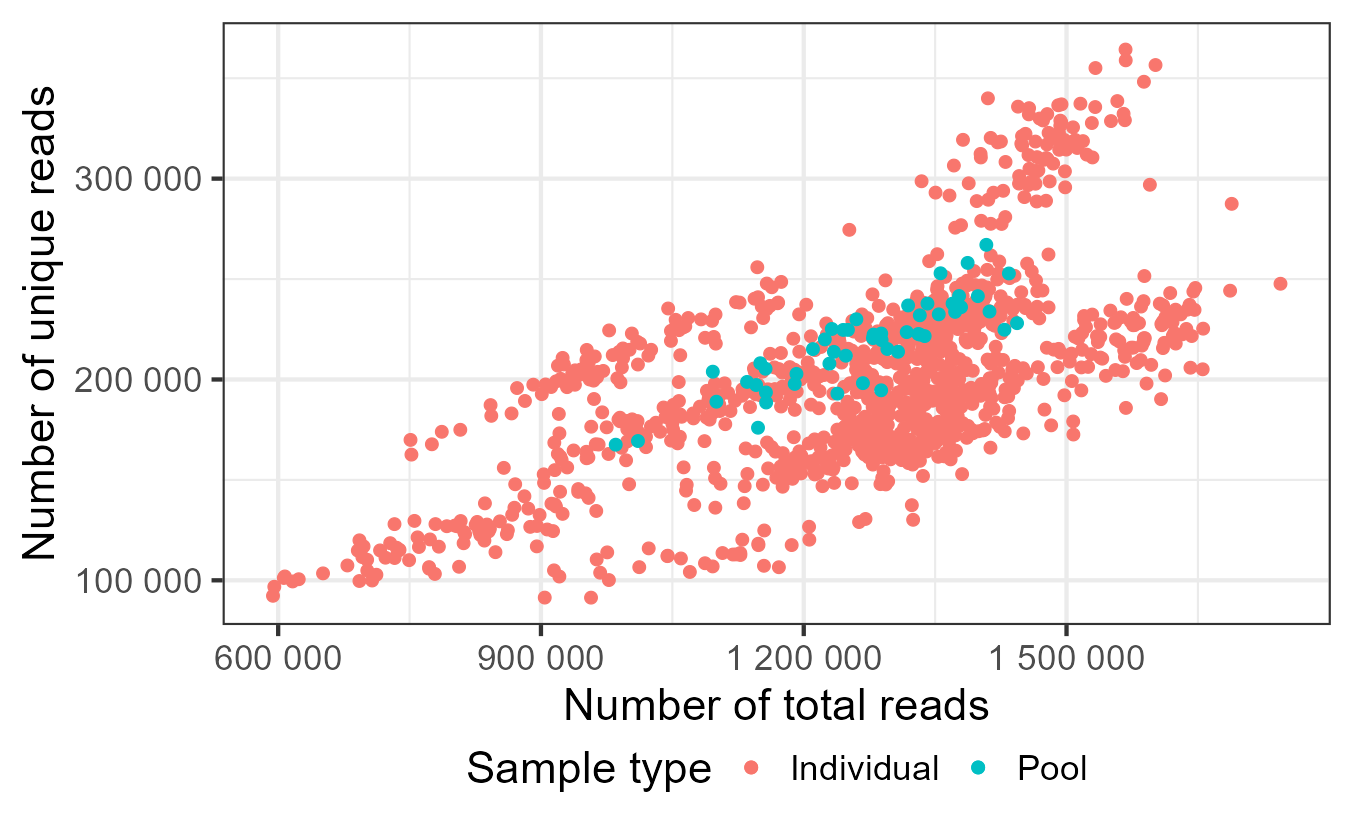


Supplementary figure 1. Distribution of total read count (X-axis) and unique read count (Y-axis) per sample, with sample types distinguished by color (individual or pool).


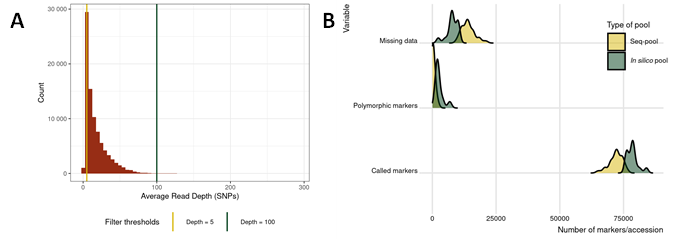


Supplementary figure 2. A. Distribution of the average read depth per SNP of the unfiltered dataset. Yellow and green lines indicate the 5 and 100 thresholds used for filtering, respectively. B. Distribution of the missing data, polymorphic markers, and effectively called markers across the unfiltered data and across types of pools.


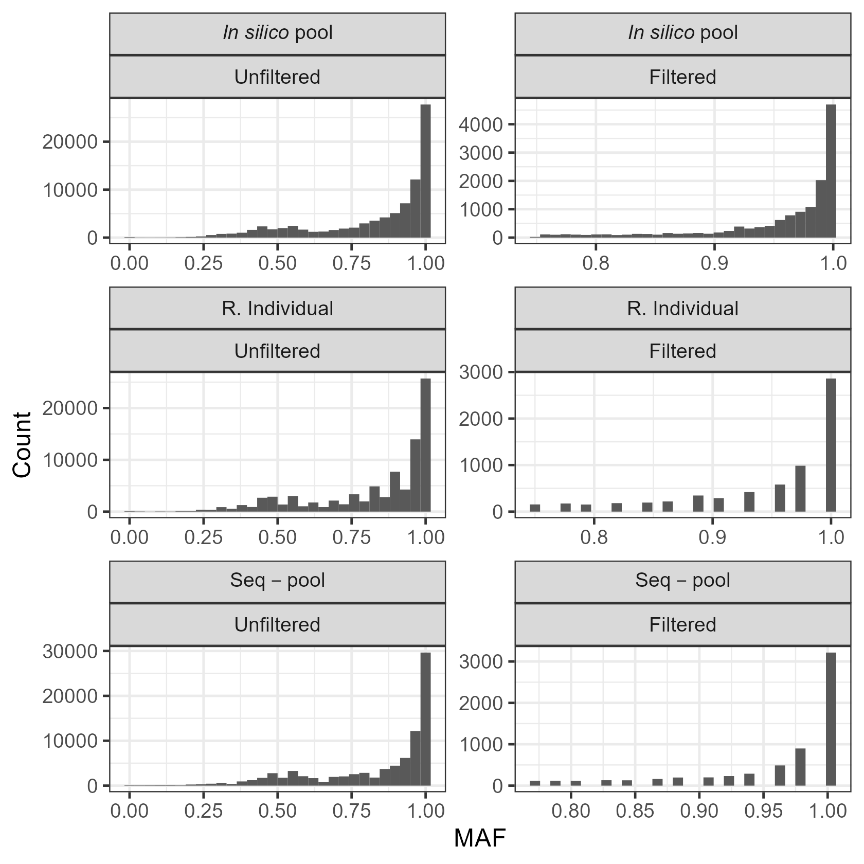


Supplementary figure 3. Histograms of the distribution of the call rate per locus across datasets, before and after filtering.


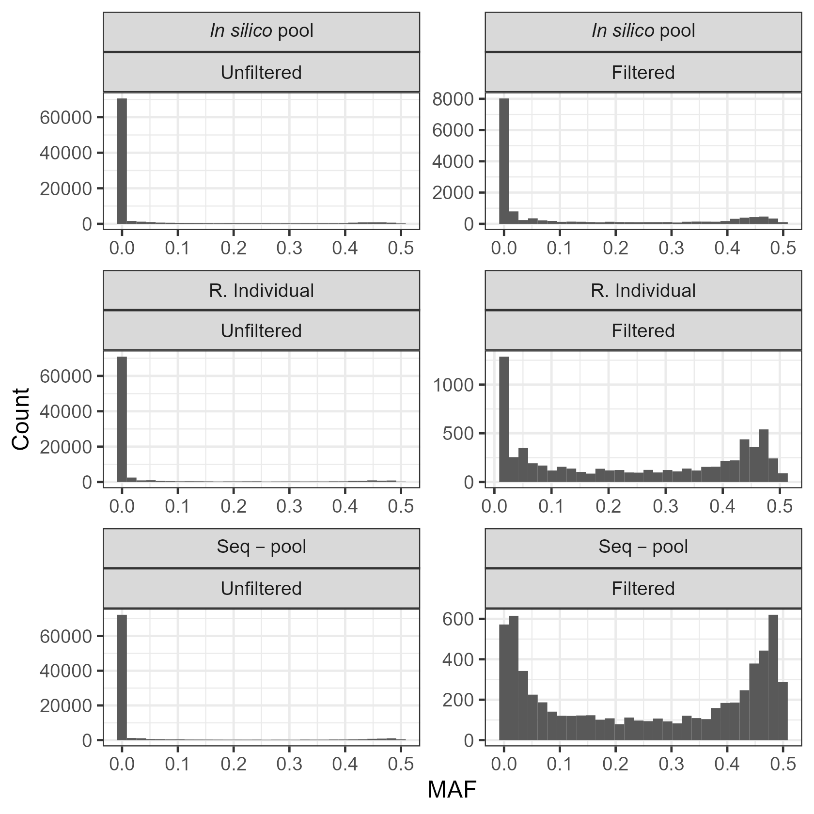


Supplementary figure 4. Histograms of the distribution of the minor allele frequency (MAF) across datasets, before and after filtering.


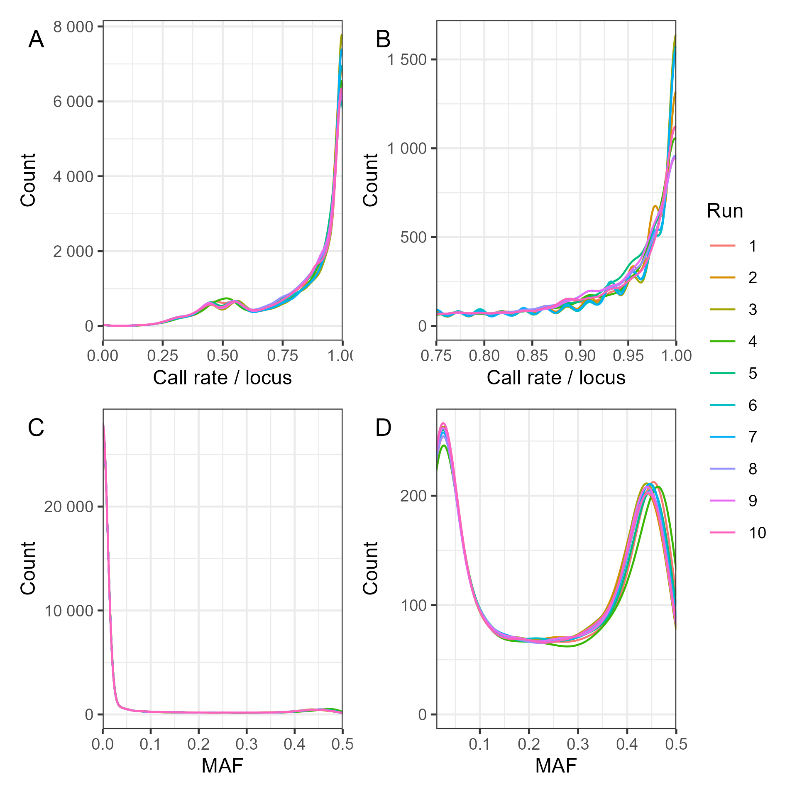


Supplementary figure 5. Distribution of summary statistics for the single plant resampling datasets. Call rate per locus before (A) and after filtering (B). Minor Allele Frequency (MAF) before (C) and after filtering (D). Each color represents a run after resampling one individual from each accession.


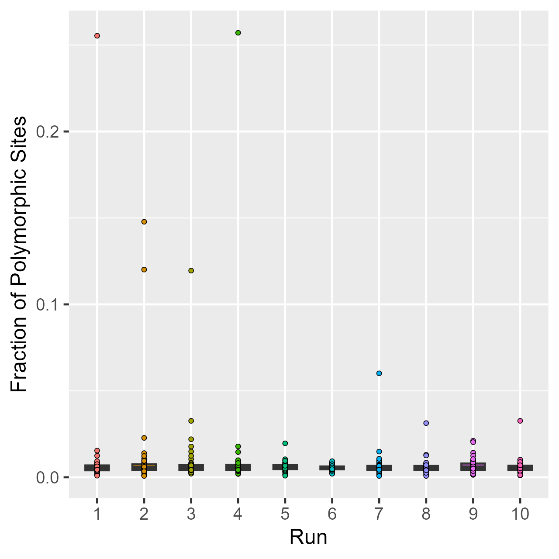


Supplementary figure 6 Distribution of the fraction of polymorphic sites per accession across resampling runs of the single plant dataset after filtering.


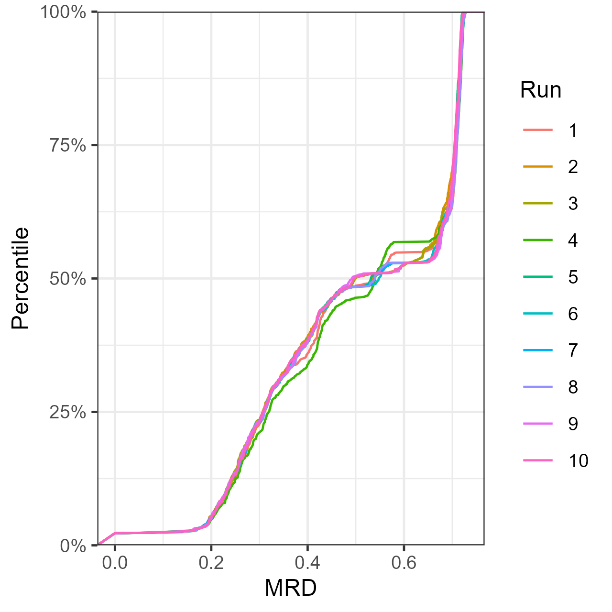


Supplementary figure 7. Distribution of the Modified Rogers Distance (MRD) across runs of the resampling runs of the single plant dataset.


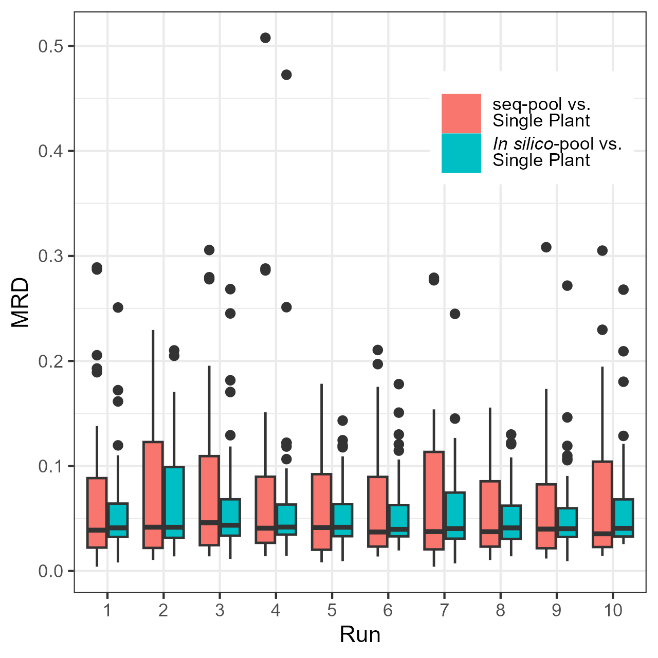


Supplementary figure 8. Comparison of the MDR between single plant samples and seq-pools (red) and *in silico*-pools (blue) of the same accession across resampling runs of the single plant dataset.


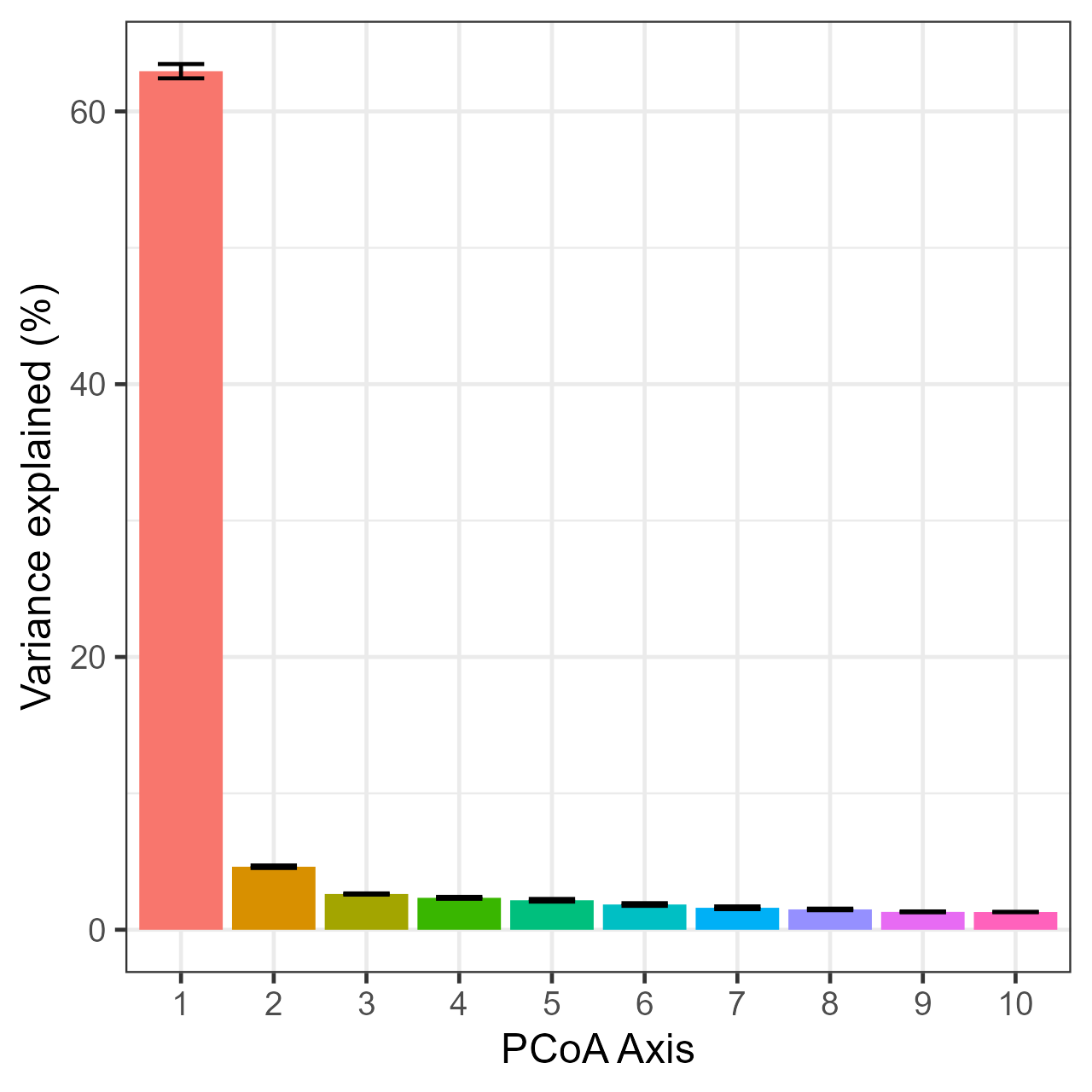


Supplementary figure 9. Percentage of the variance explained by the single plant dataset of the first ten Principal Coordinate Axes obtained from the Principal Coordinate Analysis (PCoA). Error bars indicate the standard deviation across resampling runs of the single plant dataset.


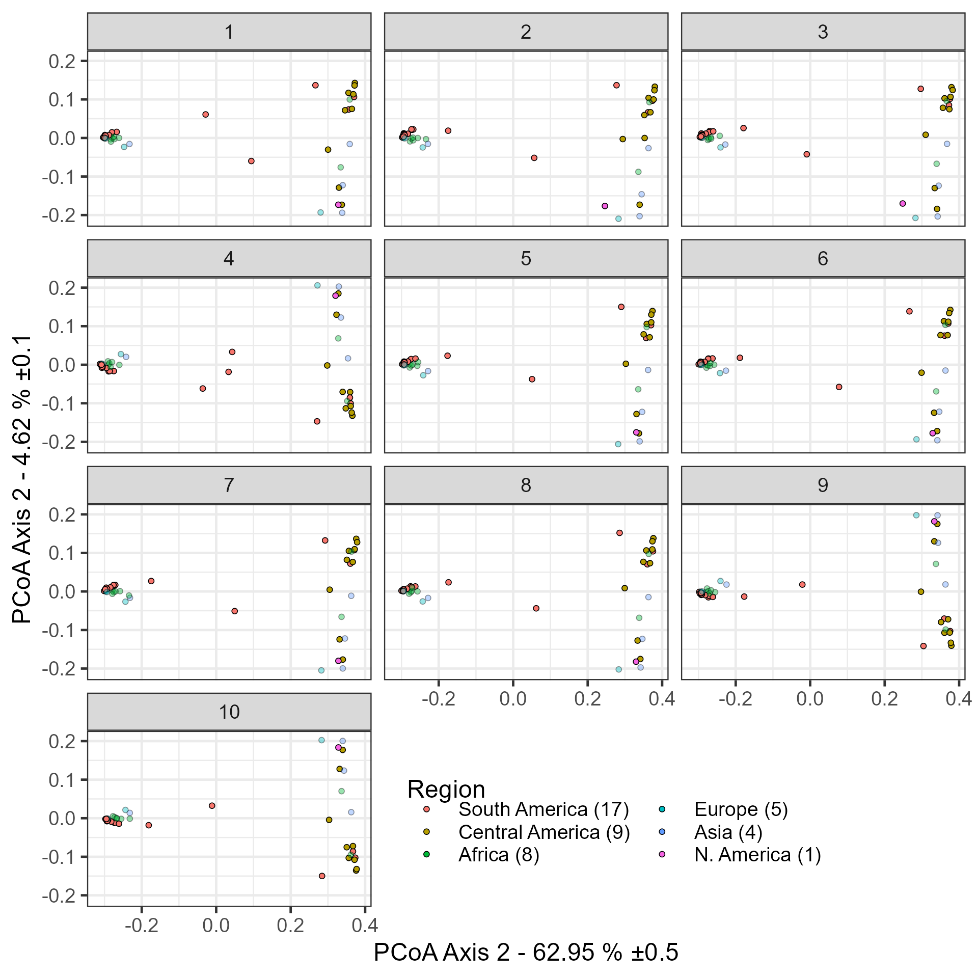


Supplementary figure 10. Scatter plot of the first two axes of the Principal Coordinate Analysis across resampling runs of a single plant per accession. Color of dots indicates region of origin. Numbers in round brackets indicate number of accessions per region.


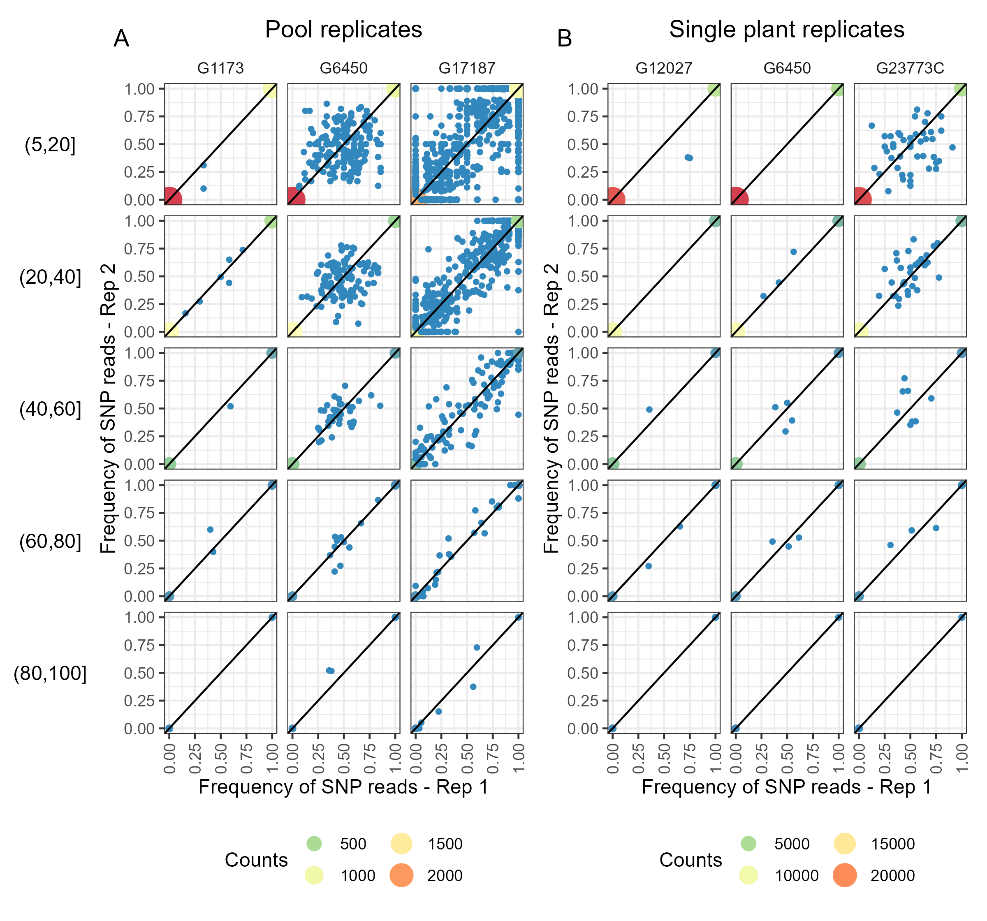


Supplementary figure 11. Comparison of SNP allele read frequencies per marker between technical replicates across different average read depth ranges (5,20; 20,40; 40,60; 60,80 and 80,100), for *seq-pools* (A) and individual plants (B). The size and color of the dots indicate marker density. Frequencies between replicates of the accessions (in columns) are shown across the average read depth intervals on the left of the figure (rows).


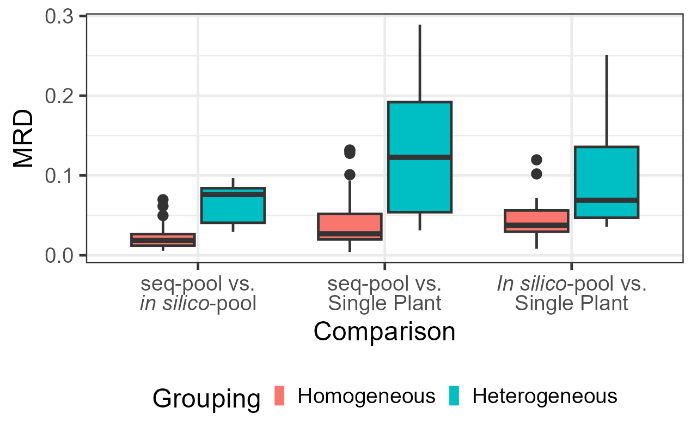


Supplementary figure 12. Boxplots of the distribution of Modified Roger’s Distances (MRD) between samples (Seq-pool, *in silico*-pool or single plant) of the same accession. Data is grouped by homogeneous (red) and heterogeneous (blue) accessions. Labels on X-axis indicate comparisons.


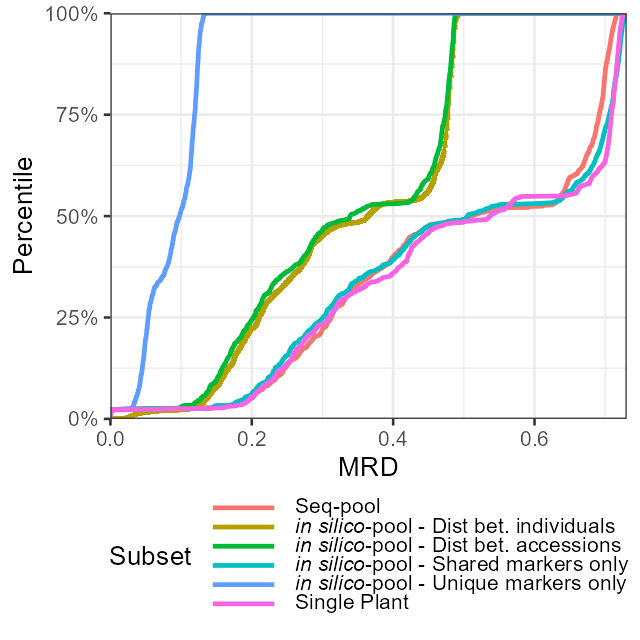


Supplementary figure 13. Distribution of the Modified Rogers’ distance (MRD) from the *seq-pool*, *in silico-pool* and single plant datasets. The MRD matrix from the *in silico-pool* was estimated as the distance between individual samples or the distances between accessions as well as from different subsets of markers, either the shared markers with the *seq-pool* dataset or the unique markers to the *in silico-pool dataset.*


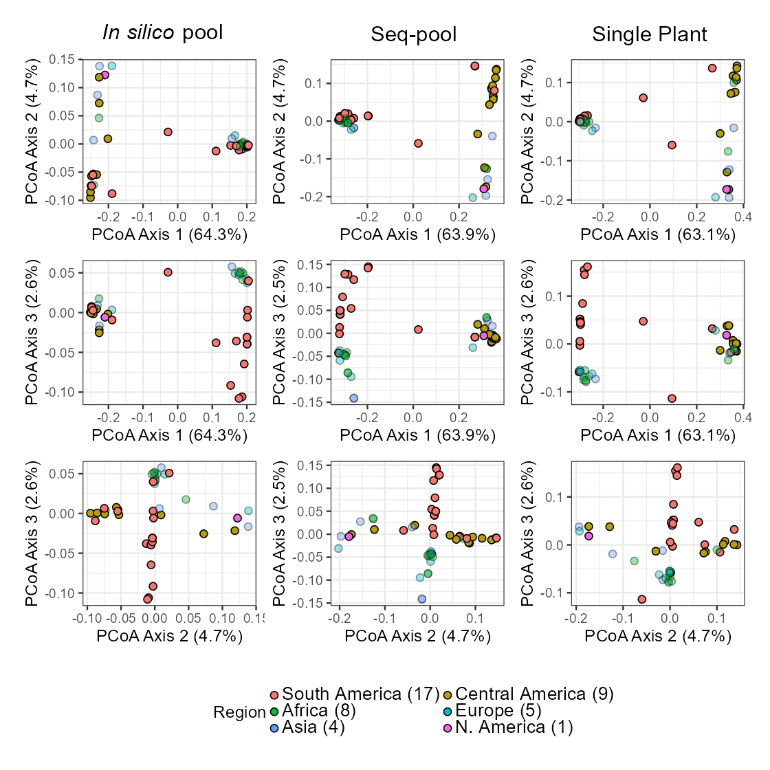


Supplementary figure 14. First three Coordinates (Axes) from the Principal Coordinates Analysis (PCoA) based on the Modified Rogers’ Distance with each dataset. Percentages in round brackets indicate fraction of the variance explained by the axis. Colors indicate region of origin according to passport data, counts in round brackets indicate frequency.


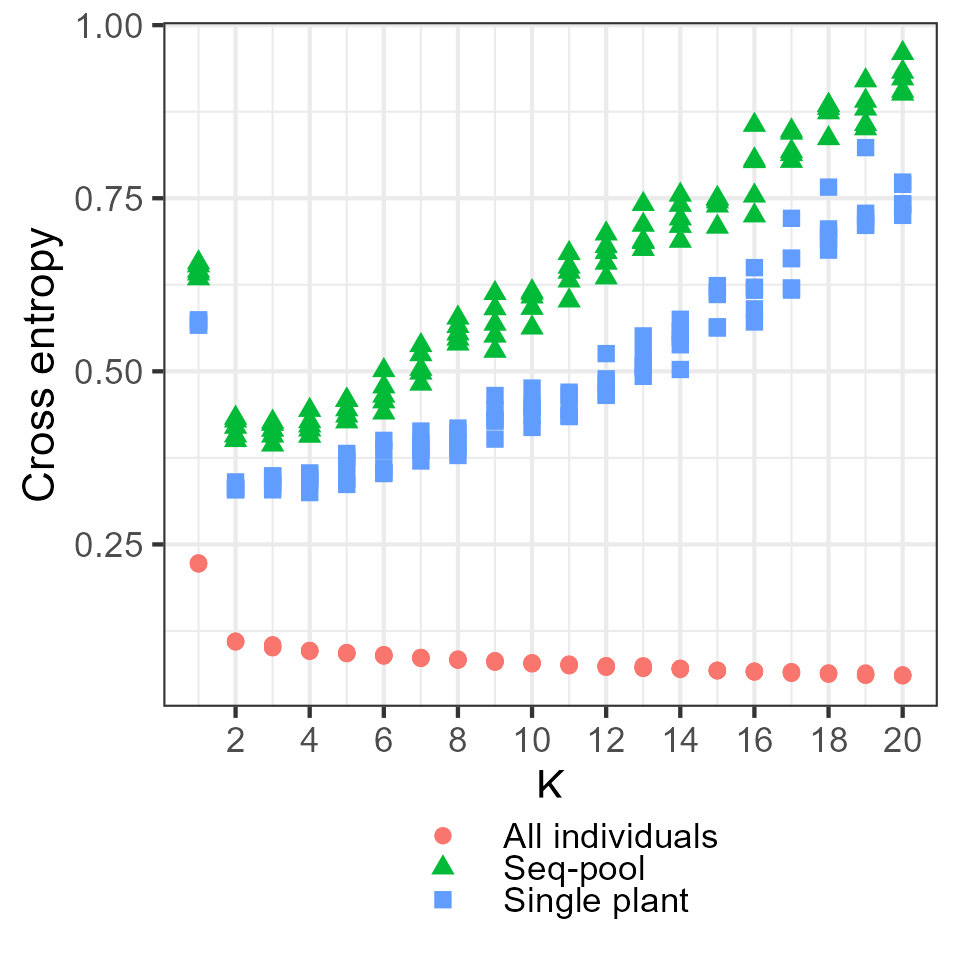


Supplementary figure 15. Cross-entropy values across datasets for multiples runs of each K ancestral populations from K=1 to K=20.


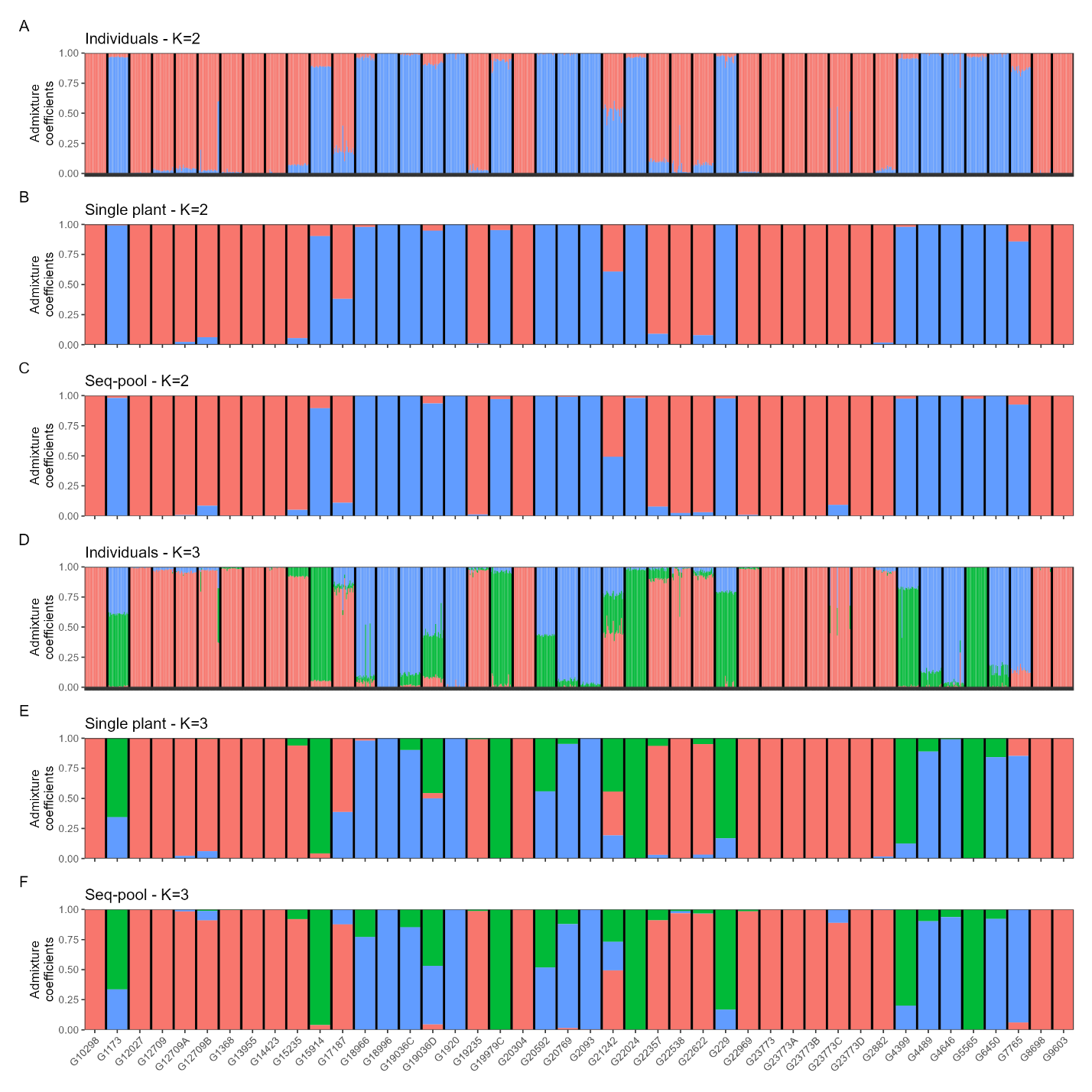
 Supplementary figure 16. Admixture coefficients from snmf across datasets and K ancestral populations.


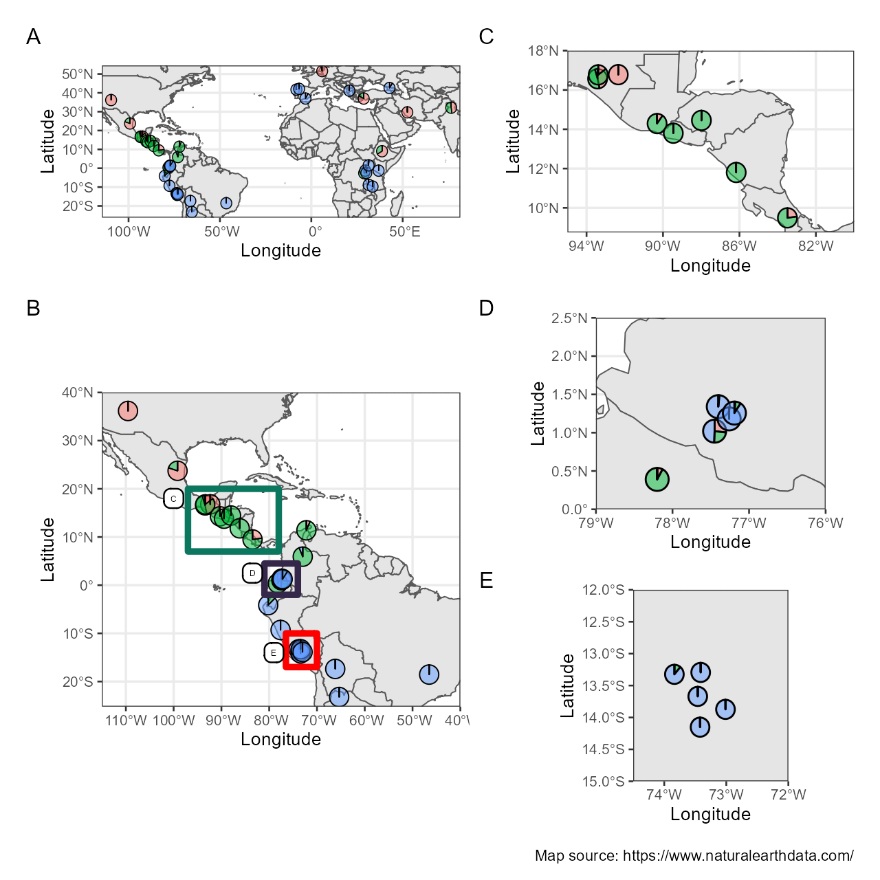


Supplementary figure 17. A-E. Admixture coefficients at K=3 from seq-pool data mapped according to coordinates of origin from the accessions’ passport data. C-E. close-ups of areas marked in B. [Passport information source: <https://www.genesys-pgr.org/a>; Map source: <https://www.naturalearthdata.com/> ]

## Supplementary tables

Supplementary table 1. Accessions used in the study, number of individuals included in the study per accession, and passport information. Passport information extracted from [genesys-pgr.org](https://www.genesys-pgr.org/).

| Accession | Number of samples | Sample | Core(1) | Country | Biological status (2) |
| --- | --- | --- | --- | --- | --- |
|  | *In silico* pool |  |  |  |  |
| G10298 | 25 | G10298 | Y | Portugal | Traditional cultivar/landrace |
| G1173 | 24 | G1173_1 | Y | Ethiopia | Traditional cultivar/landrace |
|  |  | G1173_2 |  |  |  |
| G12027 | 25 | G12027 | Y | Peru | Traditional cultivar/landrace |
| G12709 | 25 | G12709 | Y | Colombia | Traditional cultivar/landrace |
| G12709A | 25 | G12709A | Y | Colombia | Traditional cultivar/landrace |
| G12709B | 25 | G12709B | N | Colombia | Traditional cultivar/landrace |
| G1368 | 25 | G1368 | Y | Kenya | Traditional cultivar/landrace |
| G13955 | 24 | G13955 | Y | Argentina | Traditional cultivar/landrace |
| G14423 | 24 | G14423 | Y | Spain | Traditional cultivar/landrace |
| G15235 | 25 | G15235 | Y | Spain | Traditional cultivar/landrace |
| G15914 | 25 | G15914 | Y | Netherlands | Traditional cultivar/landrace |
| G17187 | 25 | G17187_1 | Y | Ecuador | Traditional cultivar/landrace |
|  |  | G17187_2 |  |  |  |
| G18966 | 23 | G18966_1 | Y | Costa Rica | Traditional cultivar/landrace |
|  |  | G18966_2 |  |  |  |
| G18996 | 25 | G18996_1 | Y | El Salvador | Traditional cultivar/landrace |
|  |  | G18996_2 |  |  |  |
| G19036C | 25 | G19036C | Y | Mexico | Traditional cultivar/landrace |
| G19036D | 25 | G19036D | Y | Mexico | Traditional cultivar/landrace |
| G1920 | 25 | G1920 | Y | Honduras | Traditional cultivar/landrace |
| G19235 | 25 | G19235 | Y | Uganda | Traditional cultivar/landrace |
| G19979C | 25 | G19979C | Y | United States | Traditional cultivar/landrace |
| G20304 | 25 | G20304 | Y | Macedonia | Traditional cultivar/landrace |
| G20592 | 23 | G20592 | Y | India | Traditional cultivar/landrace |
| G20769 | 25 | G20769 | Y | Rwanda | Traditional cultivar/landrace |
| G2093 | 25 | G2093 | Y | Nicaragua | Traditional cultivar/landrace |
| G21242 | 25 | G21242 | Y | Colombia | Traditional cultivar/landrace |
| G22024 | 25 | G22024 | Y | Mexico | Traditional cultivar/landrace |
| G22357 | 25 | G22357_1 | Y | Georgia | Traditional cultivar/landrace |
|  |  | G22357_2 |  |  |  |
| G22538 | 24 | G22538 | Y | Burundi | Traditional cultivar/landrace |
| G22622 | 25 | G22622_1 | Y | D.R of Congo | Traditional cultivar/landrace |
|  |  | G22622_2 |  |  |  |
| G229 | 25 | G229 | Y | Turkey | Traditional cultivar/landrace |
| G22969 | 25 | G22969 | Y | Malawi | Traditional cultivar/landrace |
| G23773 | 25 | G23773 | N | Peru | Traditional cultivar/landrace |
| G23773A | 25 | G23773A | Y | Peru | Traditional cultivar/landrace |
| G23773B | 25 | G23773B | N | Peru | Traditional cultivar/landrace |
| G23773C | 25 | G23773C | Y | Peru | Traditional cultivar/landrace |
| G23773D | 25 | G23773D | N | Peru | Traditional cultivar/landrace |
| G2882 | 25 | G2882 | Y | Zambia | Traditional cultivar/landrace |
| G4399 | 25 | G4399 | Y | Mexico | Traditional cultivar/landrace |
| G4489 | 25 | G4489 | Y | Guatemala | Advanced/improved cultivar |
| G4646 | 25 | G4646 | Y | Colombia | Traditional cultivar/landrace |
| G5565 | 25 | G5565 | Y | Iran | Traditional cultivar/landrace |
| G6450 | 24 | G6450_1 | Y | Ecuador | Traditional cultivar/landrace |
|  |  | G6450_2 |  |  |  |
| G7765 | 24 | G7765_1 | Y | Colombia | Traditional cultivar/landrace |
|  |  | G7765_2 |  |  |  |
| G8698 | 22 | G8698 | Y | Bolivia | Traditional cultivar/landrace |
| G9603 | 24 | G9603 | Y | Brazil | Traditional cultivar/landrace |
| (1) Part of the Bean core collection (Y: yes; N: no). | | | | | |
| (2) According to the FAO/IPGRI multi-crop passport descriptors | | | | | |

Supplementary table 2. Summary of the number of markers per type of pool and accession after filtering and comparison of sites with private alleles within each pool as well as the mean absolute Allele Frequency difference (AFD) and the percentage of homozygous SNPs within pools (Shaded rows indicate sequenced pools with technical replicates).

| Accession | Sample | Number of markers | | | | Polymorphic sites/accession | | | Private alleles | | | AFD  (1) | % homozygous SNPs within pools | |  |
| --- | --- | --- | --- | --- | --- | --- | --- | --- | --- | --- | --- | --- | --- | --- | --- |
|  |  | *in silico* pool | seq-pool | Shared markers | Single Plant | *in silico* pool | seq-pool | Single Plant | *In silico* pool | Seq-pools | Total |  | *In silico* pool | seq-pool |  |
| G10298 | G10298 | 14001 | 6145 | 5588 | 6431 | 709 | 26 | 36 | 208 | 19 | 227 | 0.0012 | 94.9% | 99.6% |  |
| G1173 | G1173 | 13659 | 5955 | 5408 | 6115 | 496 | 11 | 36 | 154 | 2 | 156 | 0.0012 | 96.4% | 99.8% |  |
|  | G1173.1 |  | 5958 | 5410 |  |  | 11 |  | 153 | 1 | 154 | 0.001 |  | 99.8% |  |
| G12027 | G12027 | 13922 | 6082 | 5528 | 6421 | 548 | 20 | 6 | 165 | 14 | 179 | 0.0008 | 96.1% | 99.7% |  |
| G12709 | G12709 | 13893 | 6026 | 5489 | 6283 | 662 | 198 | 31 | 162 | 14 | 176 | 0.008 | 95.2% | 96.7% |  |
| G12709A | G12709A | 13949 | 5922 | 5420 | 6240 | 942 | 260 | 26 | 419 | 15 | 434 | 0.0146 | 93.2% | 95.6% |  |
| G12709B | G12709B | 14070 | 6156 | 5594 | 5460 | 3492 | 1357 | 16 | 1568 | 19 | 1587 | 0.0468 | 75.2% | 78.0% |  |
| G1368 | G1368 | 13974 | 6099 | 5548 | 6397 | 677 | 27 | 29 | 191 | 20 | 211 | 0.0012 | 95.2% | 99.6% |  |
| G13955 | G13955 | 14029 | 6198 | 5619 | 6409 | 407 | 129 | 41 | 96 | 26 | 122 | 0.0035 | 97.1% | 97.9% |  |
| G14423 | G14423 | 13977 | 6157 | 5586 | 6146 | 825 | 51 | 27 | 341 | 22 | 363 | 0.0026 | 94.1% | 99.2% |  |
| G15235 | G15235 | 13915 | 5966 | 5454 | 6341 | 683 | 37 | 37 | 198 | 21 | 219 | 0.0016 | 95.1% | 99.4% |  |
| G15914 | G15914 | 13626 | 5922 | 5386 | 6186 | 778 | 40 | 47 | 211 | 29 | 240 | 0.0018 | 94.3% | 99.3% |  |
| G17187 | G17187 | 14068 | 6176 | 5608 | 6195 | 3151 | 852 | 1582 | 1760 | 17 | 1777 | 0.0302 | 77.6% | 86.2% |  |
|  | G17187.1 |  | 6166 | 5605 |  |  | 861 |  | 1751 | 19 | 1770 | 0.0302 |  | 86.0% |  |
| G18966 | G18966 | 13485 | 5972 | 5287 | 5646 | 1369 | 313 | 35 | 727 | 30 | 757 | 0.0129 | 89.8% | 94.8% |  |
|  | G18966.1 |  | 6043 | 5339 |  |  | 345 |  | 708 | 31 | 739 | 0.0112 |  | 94.3% |  |
| G18996 | G18996 | 13735 | 5853 | 5345 | 6196 | 536 | 9 | 23 | 143 | 0 | 143 | 0.0009 | 96.1% | 99.8% |  |
|  | G18996.1 |  | 6021 | 5464 |  |  | 9 |  | 149 | 0 | 149 | 0.0007 |  | 99.9% |  |
| G19036C | G19036C | 13645 | 5876 | 5342 | 6140 | 840 | 97 | 52 | 216 | 19 | 235 | 0.0038 | 93.8% | 98.3% |  |
| G19036D | G19036D | 13994 | 5688 | 5192 | 6028 | 3147 | 403 | 93 | 1665 | 18 | 1683 | 0.0255 | 77.5% | 92.9% |  |
| G1920 | G1920 | 13808 | 6059 | 5496 | 6161 | 788 | 258 | 28 | 201 | 34 | 235 | 0.009 | 94.3% | 95.7% |  |
| G19235 | G19235 | 13948 | 6129 | 5564 | 6326 | 923 | 36 | 48 | 281 | 28 | 309 | 0.0017 | 93.4% | 99.4% |  |
| G19979C | G19979C | 13794 | 5813 | 5301 | 5912 | 1929 | 63 | 37 | 1268 | 21 | 1289 | 0.0079 | 86.0% | 98.9% |  |
| G20304 | G20304 | 14000 | 6136 | 5574 | 6477 | 717 | 24 | 33 | 213 | 18 | 231 | 0.0014 | 94.9% | 99.6% |  |
| G20592 | G20592 | 13696 | 5928 | 5398 | 6240 | 372 | 32 | 9 | 107 | 19 | 126 | 0.001 | 97.3% | 99.5% |  |
| G20769 | G20769 | 13697 | 5619 | 5125 | 6228 | 836 | 34 | 42 | 214 | 19 | 233 | 0.0021 | 93.9% | 99.4% |  |
| G2093 | G2093 | 13812 | 6024 | 5472 | 6256 | 921 | 330 | 51 | 232 | 208 | 440 | 0.0108 | 93.3% | 94.5% |  |
| G21242 | G21242 | 13925 | 6071 | 5523 | 6221 | 1880 | 859 | 34 | 614 | 17 | 631 | 0.0289 | 86.5% | 85.9% |  |
| G22024 | G22024 | 13596 | 5770 | 5263 | 6076 | 928 | 40 | 50 | 326 | 26 | 352 | 0.0024 | 93.2% | 99.3% |  |
| G22357 | G22357 | 13926 | 6093 | 5532 | 6288 | 500 | 5 | 23 | 150 | 1 | 151 | 0.0009 | 96.4% | 99.9% |  |
|  | G22357.1 |  | 6090 | 5542 |  |  | 5 |  | 148 | 0 | 148 | 0.0007 |  | 99.9% |  |
| G22538 | G22538 | 14049 | 6197 | 5626 | 6409 | 2096 | 583 | 24 | 1110 | 13 | 1123 | 0.0163 | 85.1% | 90.6% |  |
| G22622 | G22622 | 13499 | 6126 | 5405 | 5677 | 526 | 4 | 25 | 217 | 0 | 217 | 0.001 | 96.1% | 99.9% |  |
|  | G22622.1 |  | 6146 | 5427 |  |  | 5 |  | 216 | 1 | 217 | 0.001 |  | 99.9% |  |
| G229 | G229 | 13591 | 5775 | 5261 | 6172 | 929 | 40 | 77 | 267 | 27 | 294 | 0.0021 | 93.2% | 99.3% |  |
| G22969 | G22969 | 13993 | 6122 | 5573 | 6455 | 672 | 57 | 46 | 170 | 48 | 218 | 0.0019 | 95.2% | 99.1% |  |
| G23773 | G23773 | 14038 | 6203 | 5625 | 6465 | 884 | 140 | 40 | 264 | 30 | 294 | 0.0044 | 93.7% | 97.7% |  |
| G23773A | G23773A | 14004 | 6187 | 5611 | 6430 | 775 | 97 | 39 | 179 | 20 | 199 | 0.003 | 94.5% | 98.4% |  |
| G23773B | G23773B | 13984 | 6162 | 5599 | 6432 | 821 | 44 | 48 | 253 | 14 | 267 | 0.0019 | 94.1% | 99.3% |  |
| G23773C | G23773C | 14071 | 6191 | 5615 | 6442 | 2974 | 1207 | 34 | 1195 | 16 | 1211 | 0.0356 | 78.9% | 80.5% |  |
| G23773D | G23773D | 14000 | 6166 | 5603 | 6437 | 727 | 83 | 60 | 167 | 11 | 178 | 0.0026 | 94.8% | 98.7% |  |
| G2882 | G2882 | 14059 | 6172 | 5605 | 6334 | 1394 | 221 | 23 | 853 | 27 | 880 | 0.0093 | 90.1% | 96.4% |  |
| G4399 | G4399 | 13982 | 5852 | 5333 | 6051 | 1600 | 123 | 40 | 927 | 17 | 944 | 0.0072 | 88.6% | 97.9% |  |
| G4489 | G4489 | 13915 | 5816 | 5322 | 6215 | 942 | 153 | 15 | 478 | 39 | 517 | 0.0069 | 93.2% | 97.4% |  |
| G4646 | G4646 | 13686 | 5927 | 5409 | 5870 | 443 | 36 | 29 | 117 | 21 | 138 | 0.0015 | 96.8% | 99.4% |  |
| G5565 | G5565 | 13601 | 5803 | 5281 | 6155 | 761 | 37 | 19 | 281 | 22 | 303 | 0.0027 | 94.4% | 99.4% |  |
| G6450 | G6450 | 13843 | 6075 | 5511 | 6179 | 709 | 346 | 9 | 103 | 0 | 103 | 0.0087 | 94.9% | 94.3% |  |
|  | G6450.1 |  | 6083 | 5515 |  |  | 342 |  | 103 | 0 | 103 | 0.0087 |  | 94.4% |  |
| G7765 | G7765 | 13923 | 6126 | 5542 | 5823 | 1693 | 805 | 35 | 473 | 22 | 495 | 0.0224 | 87.8% | 86.9% |  |
|  | G7765.1 |  | 6061 | 5496 |  |  | 709 |  | 553 | 25 | 578 | 0.0269 |  | 88.3% |  |
| G8698 | G8698 | 13988 | 6162 | 5588 | 6385 | 486 | 49 | 27 | 144 | 31 | 175 | 0.0016 | 96.5% | 99.2% |  |
| G9603 | G9603 | 14012 | 6187 | 5625 | 6455 | 394 | 30 | 20 | 109 | 19 | 128 | 0.0010 | 97.2% | 99.5% |  |
| **Mean** | | **13872.3** | **6031.8** | **5472.6** | **6209.2** | **1093.5** | **225.3** | **70.05** | **440.7** | **21.7** | **462.5** | **0.0083** | **92.2%** | **96.3%** | |
| **SD** | | **169.98** | **146.56** | **128.84** | **232.6** | **805.34** | **314.46** | **231.1** | **462.91** | **28.51** | **463.5** | **0.011** | **5.6%** | **5.3%** | |
| (1) Refers to AFD between seq-pools and *in silico*-pools | | | | | | | | | | | | | | | |

Supplementary table 3. H and H’ estimates of the expected heterozygosity (H_e_; gene diversity) per accession and type of pool and observed heterozygosity of *in silico*-*pools*. Shaded rows indicated accessions with replicated *seq-pools*.

| AccessionID | SampleID | *In silico-pool* | | *Seq-pool* | |
| --- | --- | --- | --- | --- | --- |
|  |  | **He** | | **He** | |
|  |  | H | H’ | H | H' |
| G10298 | G10298 | 0.0007 | 0.0467 | 0.000121 | 0.176685 |
| G1173 | G1173 | 0.0006 | 0.0551 | 0.000172 | 0.41336 |
|  | G1173.1 |  |  | 0.000175 | 0.419984 |
| G12027 | G12027 | 0.0005 | 0.0449 | 0.000126 | 0.195376 |
| G12709 | G12709 | 0.0024 | 0.1656 | 0.00207 | 0.349947 |
| G12709A | G12709A | 0.0028 | 0.1342 | 0.008151 | 0.255259 |
| G12709B | G12709B | 0.0128 | 0.1669 | 0.001769 | 0.376099 |
| G1368 | G1368 | 0.0006 | 0.0436 | 0.000121 | 0.2273 |
| G13955 | G13955 | 0.0015 | 0.1674 | 0.001054 | 0.343174 |
| G14423 | G14423 | 0.0012 | 0.0621 | 0.000268 | 0.252173 |
| G15235 | G15235 | 0.0007 | 0.0502 | 0.0002 | 0.23967 |
| G15914 | G15914 | 0.0008 | 0.0475 | 0.00025 | 0.205912 |
| G17187 | G17187 | 0.0096 | 0.1384 | 0.005882 | 0.328609 |
|  | G17187.1 |  |  | 0.005971 | 0.331427 |
| G18966 | G18966 | 0.0028 | 0.0818 | 0.001814 | 0.235592 |
|  | G18966.1 |  |  | 0.001787 | 0.209362 |
| G18996 | G18996 | 0.0006 | 0.0500 | 0.000121 | 0.399339 |
|  | G18996.1 |  |  | 0.000144 | 0.474888 |
| G19036C | G19036C | 0.0015 | 0.0835 | 0.000735 | 0.345945 |
| G19036D | G19036D | 0.0056 | 0.0819 | 0.003144 | 0.323788 |
| G1920 | G1920 | 0.0033 | 0.1849 | 0.002063 | 0.357885 |
| G19235 | G19235 | 0.0009 | 0.0441 | 0.000107 | 0.148021 |
| G19979C | G19979C | 0.0024 | 0.0551 | 0.000356 | 0.276459 |
| G20304 | G20304 | 0.0007 | 0.0441 | 0.000149 | 0.206615 |
| G20592 | G20592 | 0.0005 | 0.0563 | 0.000266 | 0.24187 |
| G20769 | G20769 | 0.0009 | 0.0505 | 0.000289 | 0.292625 |
| G2093 | G2093 | 0.0019 | 0.0984 | 0.002144 | 0.26169 |
| G21242 | G21242 | 0.0092 | 0.2363 | 0.00657 | 0.342365 |
| G22024 | G22024 | 0.0010 | 0.0474 | 0.00025 | 0.286834 |
| G22357 | G22357 | 0.0005 | 0.0511 | 3.4E-05 | 0.448275 |
|  | G22357.1 |  |  | 3.58E-05 | 0.472392 |
| G22538 | G22538 | 0.0049 | 0.1095 | 0.003158 | 0.239927 |
| G22622 | G22622 | 0.0006 | 0.0456 | 5.25E-05 | 0.461558 |
|  | G22622.1 |  |  | 5.33E-05 | 0.468491 |
| G229 | G229 | 0.0011 | 0.0518 | 0.00027 | 0.216128 |
| G22969 | G22969 | 0.0006 | 0.0450 | 0.000311 | 0.293195 |
| G23773 | G23773 | 0.0016 | 0.0852 | 0.000826 | 0.345649 |
| G23773A | G23773A | 0.0016 | 0.1013 | 0.000327 | 0.278318 |
| G23773B | G23773B | 0.0010 | 0.0575 | 0.006923 | 0.249359 |
| G23773C | G23773C | 0.0094 | 0.1429 | 0.000718 | 0.357386 |
| G23773D | G23773D | 0.0015 | 0.0950 | 0.000933 | 0.264398 |
| G2882 | G2882 | 0.0025 | 0.0816 | 0.001148 | 0.232754 |
| G4399 | G4399 | 0.0016 | 0.0451 | 0.000598 | 0.185602 |
| G4489 | G4489 | 0.0014 | 0.0653 | 0.000995 | 0.249878 |
| G4646 | G4646 | 0.0008 | 0.0763 | 0.000281 | 0.264469 |
| G5565 | G5565 | 0.0008 | 0.0490 | 0.000279 | 0.27291 |
| G6450 | G6450 | 0.0047 | 0.2968 | 0.003917 | 0.45303 |
|  | G6450.1 |  |  | 0.003855 | 0.445762 |
| G7765 | G7765 | 0.0079 | 0.2253 | 0.005991 | 0.344881 |
|  | G7765.1 |  |  | 0.005338 | 0.35364 |
| G8698 | G8698 | 0.0006 | 0.0557 | 0.000208 | 0.182622 |
| G9603 | G9603 | 0.0004 | 0.0500 | 0.000168 | 0.201233 |
| Mean | | 0.002 | 0.090 | 0.002 | 0.314 |
| SD | | 0.003 | 0.060 | 0.002 | 0.089 |
